# Portraying identity Mahua through emotional attachment toward Chinese Mandarin among Malaysian Chinese heritage language learners

**DOI:** 10.3389/fpsyg.2026.1681427

**Published:** 2026-04-01

**Authors:** Long Qian

**Affiliations:** School of Culture and Communication, Guilin Tourism University, Guilin, China

**Keywords:** Chinese heritage language learners, Chinese Mandarin, cultural identity, emotional attachment, Mahua

## Abstract

**Introduction:**

Since the 1990s, Mandarin has been increasingly used in Malaysian Chinese families, with the younger generation gradually shifting from viewing it as a heritage language to regarding it as their mother tongue. Against this backdrop, this study examines how emotional attachment to Mandarin (EA) shapes the cultural identity of Chinese heritage language learners (CHLLs) in Malaysian secondary schools.

**Methods:**

A mixed-methods design was employed. A total of 297 CHLLs (aged 15–18) from national and private secondary schools completed an online questionnaire. In addition, 30 participants were purposively selected for semi-structured interviews and language portrait analysis to explore their language attitudes, learning motivations, and identity narratives. The study also examined language use across family, school, and community contexts. Key demographic variables included gender, age, and school type.

**Results:**

The findings indicate that CHLLs in both public and private school systems demonstrate strong emotional attachment to Mandarin, which is widely perceived as a core marker of Chinese identity in Malaysia. At the same time, students navigate a multilingual environment in which Malay signifies national belonging, while English is associated with global mobility. Comparative analysis reveals nuanced differences: students in public schools tend to exhibit more comprehensive multilingual orientations, whereas those in private schools emphasize stronger cultural cohesion within the Chinese community.

**Discussion:**

By integrating the psychosocial model with the theory of overseas Chinese identity, this study provides a comprehensive framework for understanding how emotional attachment to a heritage language contributes to cultural identity construction among overseas Chinese learners. However, the study is limited by its regional sample scope, cross-sectional design, and the potential influence of social desirability on qualitative responses.

## 1 Introduction

Malaysian Chinese (Mahua) refers to Malaysian citizens of Han descent. It is the second largest ethnic group in the country, accounting for about 22.2% of the total population (Department of Statistics Malaysia, 2026). In the long-term multi-ethnic and multilingual social environment, the Malaysian Chinese community has continued to retain and reconstruct its ethnic traditions in terms of language, religion and cultural practice, forming a unique Malaysian Chinese culture and identity form. Historically, Chinese dialects such as Fujian dialect, Hakka dialect, Cantonese and Chaozhou Dialect were widely used within the Chinese community, while Malay and English were widely mastered to adapt to the multilingual ecology at the national level (Liao, 2020).

Since the mid-20th century, with the evolution of the national language policy and education system, Malay has gradually been established as the official language and the main teaching medium (Da Wan et al., 2020; Tan and Teoh, 2014). Nevertheless, the Chinese education system has not disappeared, but continues to exist in different institutional forms, mainly reflected in three types of secondary schools (show as Table 1): national secondary school (SMK) with Malay as the teaching medium, national Chinese secondary school (SMJK) with Chinese courses retained, and Chinese independent secondary school (STPC) with Chinese as the main teaching language (Wang, 2018). The differences in teaching language, curriculum and language ideology among different school types constitute an important institutional background for the language experience and identity construction of Malaysian Chinese students (Tan, 2011).

**Table 1:** Type of Malaysian Secondary School.

School type	Type of Malaysian Secondary School	Malay abbreviation	Medium of Instruction (MOI)	Management
Public school	National Secondary Schools	SMK, Sekolah Menengah Kebangsaan	Malay	Ministry of Education Malaysia
	National-type Chinese Secondary Schools	SMJK, Sekolah Menengah Jenis Kebangsaan	Chinese for the Chinese curriculum, but Malay for the other curriculum	
Private school	Private Chinese Secondary Schools	STPC, Sekolah Menengah Persendirian	Chinese	Dong Zong

In this context, the function of Mandarin in Malaysian Chinese society has changed significantly. It has not only become the main tool for cross dialect communication, but also occupies a dominant position in education, media and intra ethnic communication (Ho et al., 2015; Wong and Lee, 2003). To a certain extent, it has replaced the traditional Chinese dialect and become the “heritage language (HL)” of the young generation of Chinese Americans (Segawa, 2019). This change makes the language attribute of Mandarin in the Malaysian context present a certain tension: on the one hand, it is not a minority language at the national level; On the other hand, it carries the functions of ethnic memory, cultural emotion and identity symbol (Rasdi, 2020). This situation poses a new theoretical challenge to the traditional definition of “HL.”

From the perspective of sociopsycholinguistics, language is not only a communication tool, but also an important resource for identity construction. The individual’s attitude toward language, emotional input and use experience will have a profound impact on their ethnic affiliation and cultural identity (Kelleher, 2010; Süleyman, 2024). Based on Gardner and Lambert (1972)’s psychosocial model (Rosiak, 2023), this study focuses on emotion, motivation and social environmental factors in language learning; At the same time, Wang (1988)’s overseas Chinese identity theory is introduced to emphasize that the overseas Chinese identity is a process of dynamic negotiation and reconstruction in a multilingual and multicultural context. By integrating the above two theoretical perspectives, this study attempts to construct an analytical framework to explain the relationship between Mandarin emotional attachment and Malaysian Chinese identity of Malaysian CHLLs.

Existing studies mostly focus on the language maintenance, language transfer or macro identity issues of Chinese Malaysians, but pay insufficient attention to the differences of emotional attachment (EA) and its psychological mechanism formed by CHLLs in different school systems, especially the lack of empirical research that systematically compares emotional factors, learning motivation and school types (Zhou, 2016; Kondo-Brown, 2003). In view of this, this study takes the CHLLs in national middle school (SMK/SMJK) and Chinese independent middle school (STPC) as the object, uses the mixed research method, and compares and analyzes how different educational situations shape the learners’ emotional attachment path to Mandarin and the construction of their cultural identity:

1 What are the shared characteristics and differences of Language Input and Choices in family and educational domains among the CHLLs between public and private schools?
2 What are the shared characteristics and differences in the Language Learning Process (Attitudes and Motivations) toward learning Mandarin among the CHLLs between public and private schools?
3 What are the shared characteristics and differences in Language Learning Experience through Language Portraits of CHLLs between public and private schools?
4 How do the CHLLs between public and private schools construct their Cultural Identity in a multilingual Malaysia context?

## 2 Literature review

### 2.1 The language and identity of the Malaysian Chinese (Mahua)

The term Chinese or Huaren is an exonym for Malaysian Chinese (without taking into consideration the context of linguistic varieties). Within their own spoken language group, various designations such as Hokkien, Cantonese, Hakka, Foochow, Henghua, and others have been used as endonyms, and all these endonyms have a connection to the historical inheritance of identity. The process of regionalization, however, has altered this historically held heritage identity, and over time, the Chinese community in Malaysia has assimilated into Malaysian society while maintaining their distinct cultural identity, with some experiencing more localization to the extent that they have developed a new view of Chinese identity (Khoo, 1999). This “extended localization” of Chinese identity refers to the Baba and Peranakan Chinese (Khoo, 1999). Indeed, scholars’ interest in studying Malaysian Chinese has traditionally concentrated on the themes of cultural assimilation and identity among the Baba and Peranakan Chinese; nevertheless, compared to Baba and Peranakan studies, those focusing on “pure Chinese” are somewhat less popular. This is probably due to the perception that their identity is less outstanding than that of the Baba and Peranakan Chinese. In reality, various identity issues have been identified within the so-called “pure Chinese” community. One of these is the identity developed through the use of Mandarin Chinese in Chinese-medium schools and as the Chinese’s family language. As previously stated, the Chinese in Malaysia speak their ancestral Sinitic languages, including Hakka, Hokkien, Cantonese, Foochow, Teochew, and others. However, for present-day Chinese youngsters, Mandarin Chinese has gradually replaced the languages of their ancestors, leading to changes in the linguistic patterns of Chinese society (Yao, 2021).

As a result of constant exposure to Mandarin Chinese, an increasing number of Chinese teenagers are unable to communicate in their parents’ native language; Mandarin Chinese is frequently used by them with their parents at home and in daily intra-ethnic interactions in both formal and informal settings (How et al., 2015). However, the phenomenon of mother tongue shifting in the Chinese community raises issues regarding the young Chinese’s motivation and attitude toward their parents’ first language and Mandarin Chinese. In this circumstance, do the younger generations see Mandarin Chinese as their “roots” and a sign of Chinese identity, as previous generations did with their mother tongue? The scenario has become more complicated since a growing number of Chinese parents’ desire to converse with their children in English (Zou, 2018).

### 2.2 Emotional attachment (EA) toward heritage language

In terms of EA, attachment to the community’s language reshapes the speakers’ identities and helps in the preservation of their language (Hatoss and Sheely, 2009; Gomaa, 2011; Benrabah, 2014). EA to the language itself may lead to fear of losing the language since it represents identity, and failing the language can lead to identity loss (Stoessel, 2002). The EA of Malaysian Chinese to Mandarin Chinese, also referred to as “mother tongue awareness” by the Chinese community, can reflect both the identity of the language speaker and the depth of their self-acquisition (Zhang, 2015). The Chinese’s EA (especially among the younger generation) to Mandarin Chinese may be reflected in this sense of mother tongue, cultural, and traditional values. However, empirical studies on EA to Mandarin Chinese language and Mandarin Chinese as a HL are very limited, and comparative studies across other overseas Chinese contexts, such as the United States or Australia, are scarce (Abdelhadi, 2018).

The study of Mandarin Chinese as a HL is an emerging field in Applied Linguistics, as previous research focused mainly on learning Mandarin Chinese as L2 or FL (Montrul, 2016). According to reviews of the literature, studies on Mandarin Chinese as HL or L1 are mainly focused on educational issues (Samuel and Tee, 2013), with little research on the micro level, such as language awareness and motivation (Sua and Santhiram, 2017). Furthermore, previous research on the motivation to learn Mandarin Chinese is very limited in the Malaysian context compared to studies in Anglophone countries. For example, Zhang et al. (2024) report that there have been such studies conducted in the US, Australia, and Canada, respectively. Moreover, most of these existing studies lack an interdisciplinary approach that integrates perspectives from psychology, sociology, and linguistics.

Based on this, this study regards emotional attachment as a multi-dimensional psychological construction at the theoretical level, including emotional identity, value evaluation and behavior tendency. It should be pointed out that this study does not presuppose the specific structure of emotional attachment, but through exploratory factor analysis (EFA) of quantitative questionnaire data, 10 potential dimensions are summarized on the basis of empirical data. These 10 dimensions reflect the structural characteristics of Malaysian Chinese heritage language learners’ emotional attachment to Mandarin, including emotional identity, cultural symbolic meaning, language belonging, value identity, social function cognition and so on. The extraction of factor structure is based on statistical criteria (such as eigenvalue greater than 1, factor load threshold, etc.), and its reliability is verified by internal consistency test (see the following research methods for details).

Therefore, based on previous theories, this study interprets EA as a dynamic psychological structure formed in a multilingual society, whose specific dimensions are affected by the social system environment, language ecology and individual experience. The 10 dimensions of emotional attachment identified in this study were derived from the exploratory factor analysis (EFA) of the self-administered questionnaire. These dimensions represent different aspects of learners’ emotional connection with Mandarin, including identity symbols, cultural affiliation, communicative value and emotional resonance. This multi-dimensional conceptualization is consistent with previous studies (Pavlenko, 2005; Dewaele, 2010; Panicacci, 2019; Liu, 2025), which emphasize that emotional attachment to language is closely related to heritage language maintenance, identity construction and socio-cultural attribution in a multilingual environment. In the context of the coexistence of multilingual and multi education systems in Malaysia, learners’ emotional attachment to Mandarin may not only strengthen ethnic identity, but also be affected by mainstream languages and global languages (such as Malay and English) and present a complex negotiation state (Abdelhadi, 2018). In general, the existing studies have confirmed the important role of emotional attachment in language maintenance and learning motivation, but in the multilingual context, whether language necessarily constitutes the core of identity, how different education systems affect the structure of emotional attachment, and whether emotional attachment further affects the motivation and identity orientation of the new generation of learners remain to be discussed. This study is based on the above theoretical and empirical gap.

### 2.3 A review of CHLL in global contexts

When considering scenarios beyond Malaysia, research on Chinese as HL in other countries with significant Chinese diaspora communities, particularly in the Anglophone countries like US, Canada, and Australia, has indeed seen a flourishing focus on CHL education in recent decades (Zhang et al., 2024). In his 2023 article, Zheng (2023) explores how Chinese as HL shapes the identity of the Chinese diaspora in the United States. He argues that CHL is more than just a language but it also a core part of how heritage identity is formed and maintained. Zheng’s analysis focuses on three main areas, i.e., CHL education, proficiency, and family influence. Regarding the role of CHL education, Zheng focuses on community-based heritage language schools. He argues that these institutions serve as crucial sites where language and culture are formally taught, enabling learners to connect with their heritage. However, these schools sometimes present a narrow view of Chinese culture and may not reflect the diverse, transnational experiences of students. For CHL proficiency, Zheng notes that proficiency enables deeper engagement with cultural practices and community networks, whereas limited ability can lead to disconnection and identity ambivalence. The shift toward English dominance among second-generation immigrants further complicates this, often resulting in heritage language attrition and weakened cultural affiliation. Finally, he highlights the family as the primary domain for CHL transmission. Parental attitudes and home language policies significantly influence children’s bilingual development and bicultural identity. The practice of using Chinese at home strengthens identity formation, while conversely, an English-only approach can alienate younger generations from their heritage.”

Another study by Wen (2024) in US investigated the motivation of CHL learners with diverse Chinese language backgrounds at an American university, focusing on the factors that motivated them to enroll in and persist with Chinese courses. The study revealed several key findings. First, compared to non-heritage learners, CHL learners spent significantly more time on course-related work, indicating a stronger academic commitment. Second, learners’ motivation was dynamic and evolved through interactions with family, teachers, friends, and the community. Pivotal experiences, such as family gatherings, classroom challenges, or community involvement, shaped their identities and reinforced their commitment to learning Chinese. Finally, a core motivator was the learners’ desire to connect with family members and affirm a Chinese cultural identity. This intrinsic, culturally rooted drive often interacted with and strengthened other motivational factors.

In Australia, a study by Huang and Liao (2023) also investigated a topic related to Chinese as a HL. It examines how Chinese–English interlingual families in Australia maintain Chinese as a heritage or minority language with their preteen children (aged 10–12), focusing on their attitudes, practices, and challenges. The study found that Chinese parents viewed Chinese as essential for cultural heritage and identity and believed it offered future career benefits. As more than one-third of registered marriages in Australia are interethnic, some parents avoided speaking Chinese to prevent isolating the non-Chinese-speaking spouse and to maintain family harmony. Similar to the situation in the U. S., community-based heritage language schools are popular in Australia. These schools have become a common choice for Chinese immigrant parents to send their children to learn the language and maintain their heritage. In these community schools, children receive structured language input, literacy development, social opportunities, and motivation. However, their effectiveness depends on family engagement, and a lack of involvement reportedly leads to lower motivation and proficiency. From the perspective of language use and proficiency, consistent parental use of Chinese at home correlated with higher child proficiency, especially in speaking and listening. Children with limited exposure or low confidence tended to respond in English.

In Canada, a multilingual and multicultural country with large numbers of visitors and immigrants, the Chinese who are also a major immigrant group encounter the dynamics of Chinese as a HL, influenced by competition with dominant languages such as English and French. Indeed, the maintenance and study of heritage languages have emerged as critical areas of educational and sociocultural inquiry. To understand this phenomenon, Chen (2024) investigates the complex ways in which language ideology shapes the motivations and educational journeys of university students learning Mandarin as a heritage language. The study is guided by three central research questions. First, it seeks to understand how learners experience Mandarin acquisition across their lifetimes, from childhood to university. Second, it identifies the primary motivations that drive these individuals to pursue formal Mandarin study at the post-secondary level. Finally, it examines how language ideologies influence both their motivations and their learning experiences. The findings reveal that childhood encounters with Mandarin education were overwhelmingly characterized by coercion, disengagement, and negative affect. Participants recounted being forced by parents to attend weekend “Chinese school,” where pedagogy focused on rote memorization of ancient poetry and character writing—content they found irrelevant and “pointless.” Conversely, their university-level Mandarin courses were described positively. Learners appreciated the practical, communicative focus and the supportive resources available to them. Regarding motivation, the most universal motivator was the desire to build and deepen familial and social connections, particularly with grandparents and relatives who are monolingual Mandarin or Cantonese speakers, or a sense of cultural obligation. Instrumental benefits, such as enhancing career prospects in international business was also significant. Furthermore, personal interest in Chinese culture and media (including social media platforms) served as additional motivation. Concerning motivations related to language ideology, Chen’s analysis demonstrates that ideologies positioning Mandarin as a global language of economic power and English as the dominant Canadian lingua franca influenced the learners’ priorities. At an institutional level, educational practices that privileged Mandarin over other Chinese dialects reinforced the idea of Mandarin as the “legitimate” Chinese language. At the micro level of family, ideologies connecting language proficiency to cultural authenticity and familial duty were powerfully internalized. In summary, this study demonstrates that heritage language learning is deeply personal journey of connecting with family, negotiating identity, and navigating the powerful social currents that ascribe value to one language over another. Despite the research gaps identified in sections 2.1 and 2.2, a review of studies on CHLL in the United States, Australia, and Canada reveals that learners typically acquire Chinese through informal education, specifically in community or weekend schools. In contrast, Chinese is taught within the mainstream educational system in Malaysia. This contrast reveals a significant research gap that needs to be addressed: the EA related to the Chinese language among students in Malaysia who have studied Chinese in national mainstream education (SJKC) for at least 6 years, many of whom continue their formal education in Mandarin at STPCs. The findings from this context could provide valuable comparative insights for the field of CHLL studies.

## 3 Methods

This study adopts sequential exploratory mixed methods design. The research first explores the constituent elements of the emotional attachment (EA) of Malaysian CHLLs to Mandarin through qualitative methods. On this basis, the quantitative measurement tool is developed and revised, and verified and compared in a larger sample. The integration of the research is reflected in three levels: first, the qualitative results are used to generate and revise the scale entries; Second, the potential structure was verified by exploratory factor analysis; Third, at the interpretation level, the quantitative results are supplemented with qualitative interview materials. The interpretive integration of quantitative and qualitative data facilitated a comprehensive analysis. Quantitative data from 297 respondents revealed generalizable patterns in language use, attitudes, and EA levels across different school types. These trends were subsequently elucidated and enriched by contextualized narratives derived from 30 in-depth qualitative cases. The design’s principal strength is its capacity to answer interrelated questions: the quantitative data delineates the “what” and “how much,” while the qualitative inquiry explores the “why.” For example, where quantitative scales for “Chinese Cultural Identity” identified specific constructs, qualitative language portraits gave them meaning by explaining their personal significance, such as associating dialects with the “abdomen” to represent a connection to ancestral heritage (See the example in section 4.3.4).

### 3.1 Participants and sampling

The study uses stratified purpose sampling to select three types of secondary schools in Penang, Johor and Sarawak, Malaysia, including national secondary school (SMK), national Chinese secondary school (SMJK) and Chinese independent secondary school (STPC). The sample selection criteria are: the participants must be Malaysian Chinese students, aged between 16 and 18, who have received Chinese education since the first grade of primary school, and have participated in the primary school standard curriculum examination (UPSR). Non-Chinese students, non- Chinese native speakers and those who did not take the UPSR examination were excluded.

A total of 320 questionnaires were collected in the quantitative stage, and 297 valid samples were screened. There were 125 males (42.1%) and 172 females (57.9%), while 151 participants in the public school group (SMK and SMJK) and 146 participants in the private school group (STPC). In the qualitative stage, a total of 30 students were selected to participate in the language portraits and in-depth interview, with 10 students from each type of school. The sample is almost balanced in gender and grade (12 males and 18 females; 16 senior 4 and 14 senior 5). Participants were screened according to the initial test results of emotional attachment and expression ability to ensure the diversity and representativeness of the sample. Data collection reached theoretical saturation when three consecutive interviews did not produce new topics.

### 3.2 Qualitative phases

The study used the language portrait task to guide participants to mark different languages on body images and explain their symbolic meanings, so as to deepen the understanding of the symbolic level of language emotion. The qualitative part of the interview was conducted in Mandarin, with an average length of about 60 min. The semi-structured interview outline was used to focus on the following core issues: the subjects’ cognition, attitude and motivation to learn Chinese; Identification and emotional attachment to Chinese culture, the impact of school environment on language emotion, and the relationship between Mandarin and identity. All interviews were recorded and transcribed into text.

#### 3.2.1 Data collection instruments

##### 3.2.1.1 Language portraits

In this study, the language portrait approach was combined with interviews to survey three groups of participants from SMK, SMJK, and STPC (about 10 in each group). Before the language portraits survey, the participants are requested to prepare a sheet of paper and colored pens. At the beginning of the survey, participants were asked a few basic demographic questions, including their age, the type of school they attended, the language they spoke, and when they first started learning Mandarin. After that, they are required to draw a silhouette and are instructed to fill in various parts of the body with different colors, each color representing a language that they choose, either a language they currently speak, a language they would like to learn, or their most preferred language.

##### 3.2.1.2 Online semi-structured interview

After the participants finished drawing their language portraits, the three groups of subjects were interviewed using an interviewing guide developed based on previous studies on language portraits (Prasad, 2014; Busch, 2016; Busch, 2018; Soares et al., 2020). The interviews were conducted in Mandarin, as this is the language of preference for the participants. The interviews were recorded on a virtual platform (Zoom), and the gathered material was accurately transcribed. In addition, the participants were asked some in-depth questions about motivation, attitude, EA and the relationship between EA and motivation and attitude.

#### 3.2.2 Data analysis

The data analysis in the qualitative stage systematically processed the language portrait and semi-structured interview materials. Firstly, the language portrait data adopts the visual narrative integration analysis path: the researcher’s number and scan all the images, record the spatial distribution of different languages in the body parts, and conduct open coding and mode induction according to the symbolic meaning of the body. At the same time, combined with the participants’ oral interpretation of color and position selection, the theme analysis is carried out to identify the symbolic relationship between language and emotional attachment. Secondly, the interview data were transcribed word for word and processed by reflective topic analysis, including line by line open coding, concept aggregation and topic extraction, focusing on the identification of meaning structures related to emotional attachment, identity and learning motivation. The two researchers independently coded part of the samples and calculated the consistency coefficient (Cohen’s *κ* > 0.80). When there were no new topics in the continuous interviews, they were judged to reach theoretical saturation. Finally, by integrating visual and textual data through matrix comparison, the core dimensions of emotional attachment are extracted and used as the theoretical basis for the subsequent scale construction.

Qualitatively, EA was measured using two representative methods: language portraits and semi-structured interviews. In the language portrait activity, participants mapped their linguistic repertoire onto a body silhouette, using this metaphorical framework to express the emotional and identity-based significance of each language. The subsequent semi-structured interviews then explored participants’ personal experiences, emotions, and narratives related to Mandarin, focusing on its personal significance, its role in familial and cultural connections, and the emotional responses it elicits. Through the language portraits method, individuals can map their emotional hierarchy toward languages, using spatial placement to symbolize attachment. For example, positioning a language over the heart to represent love and deep affection. The accompanying interviews provide essential narrative context for these visual abstractions, allowing researchers to explore the stories behind the colors and placements, as well as the personal meaning of this linguistic attachment.

### 3.3 Quantitative phases

The sociolinguistic survey in quantitative phrase that includes questionnaires on (a) demographics, (b) language background and language use in daily life, (c) motivations and attitudes for learning Chinese (adapted from AMTB, shown as 3.3.1) and (d) EA and identity.

To ensure the effectiveness of the questionnaire, a pilot study was conducted online from July to August 2022. Two Malaysian Chinese undergraduates assisted in contacting national and private secondary schools across several states, and with teachers’ support, 45 questionnaires were distributed via Google Form. A total of 45 students (16 males, 29 females, aged 15–25) participated, providing responses as well as suggestions and comments to refine the instruments.

#### 3.3.1 Data collection instruments

The questionnaires used in this survey are divided into four sections:

*The demographic section*: Section A contains demographic questions designed to determine the different types of groups that participated in this study. Section A requested information about each participant’s age, ethnicity, gender, residence, grade level, and other relevant details. According to Park et al. (2012) self-reported demographic questionnaire has certain advantages since “it can provide information from a large number of participants and can be objectively compared and interpreted through statistical data analysis”.

*Language backgrounds and language uses*: The questions in Section B can be summarized as inquiries about the participants’ language backgrounds and the languages they use in their daily lives. This part includes respondents’ language use in family and education domains, based on Fishman’s domain and language choice (Fishman, 1972).

*Language Attitude, Motivations Test Battery (AMTB) Questions*: The 55 items in Section C are designed to study the learning motivations and attitudes of Mandarin as a HL by adapted from Gardner and Lambert’s “Attitude Motivation Test Battery” framework (Gardner et al., 1985; Masgoret and Gardner, 2003). The reason for adopting AMTB is that it has been used in numerous qualitative and quantitative studies that have examined various affective components influencing second/foreign language learning (Masgoret and Gardner, 2003; Williams et al., 2002). In addition to being widely used in studies on second and foreign languages, AMTB has also been employed to investigate the motivation and attitudes of HLLs toward learning a language. For example, according to Noels (2005)’ study, HL students exhibit significant integrative and instrumental motivation and are more intrinsically motivated than L2 learners (Comanaru and Noels, 2009). Gardner and Lambert divide the AMTB into three main sections: (a) attitudes (e.g., I really enjoy the progress of learning Chinese.), motivations (e.g., Studying Chinese can be important for me because I want to do well in Chinese, it is natural for me.), and classroom anxiety (I never feel quite sure of myself when I am speaking in our Chinese class.); (b) motivational intensity (e.g., I actively think about what I have learned in my Chinese language class.); and (c) teacher and curriculum. However, the questions developed for this study are limited to the AMTB’s sections (a) and (b) (Gardner et al., 1985).

*Identity and EA questions*: The 78 items of Section D scale were adapted to the Malaysian setting and include the themes summarized in the qualitative stage, as well as adapted from Zhang’s (2011) Questionnaire on the Ethnic Identity of Filipino-Chinese Secondary School Students and the questionnaire developed by von Scheve et al. (2014) to measure national identification. The two-way translation procedure is adopted for the translation of items: first, the English items are translated into Chinese, and then back translated by bilingual experts, which are reviewed by the expert group. Then a pilot study was conducted to revise the wording and expression according to the feedback. Finally, the emotional attachment scale with 78 items was formed, covering 10 dimensions and 7–8 items in each dimension. The five-point Likert scale was used for scoring (1 = very disagree, 5 = very agree).

#### 3.3.2 Reliability and validity of the sample distribution

This subsection presents the reliability and validity analysis of the sample data. As detailed in the Appendix A, the “AMTB” scale’s identity & EA component shows exceptionally high internal consistency. The total Cronbach’s alpha for the scale was 0.938, indicating excellent reliability for the measurement of the overarching identity and EA constructs. Furthermore, all subscales also show high reliability, with alpha values ranging from 0.845 to 0.953. For example, Malaysian Cultural Identity (K1-K4): *α* = 0.845 (e.g., It is important for us Malay citizens to understand the Malay cultural traditions.); Chinese Cultural Identity (L1-L10): *α* = 0.952 (e.g., I believe that the customs of the Chinese community should be preserved and heritage.); Tionghua Traditional Cultural Values (M1-M6): α = 0.928 etc. (e.g., I think the Chinese community should celebrate the traditional Chinese festivals: the Chinese New Year, the Lantern Festival, the Mid-Autumn Festival and the Dragon Boat Festival.).

In terms of validity, the author evaluated the sample’s suitability for factor analysis using the Kaiser-Meyer-Olkin (KMO) measure and Bartlett’s Test of Sphericity (see Appendix B) (Kaiser, 1974). The resulting KMO value of 0.883 is considered excellent, indicating compact correlations between items and confirming that the data is highly appropriate for factor analysis.

The construct validity of the scale was assessed using the Rotated Component Matrix of EA (Appendix D). The analysis revealed a clear and interpretable factor structure, indicating that nearly all items load strongly (typically > 0.7) on their intended components with minimal cross-loading. For instance, National Identity items (J1-J12) predominantly loaded on Component 2, while EA to Customs items (N1-N13) loaded on Component 1. These results confirm that the scale effectively measures the multiple unique dimensions of identity and EA.

#### 3.3.3 Data analysis

This study utilized a mixed-methods approach to comprehensively measure EA. SPSS software was used for data analysis. Firstly, descriptive statistical analysis was carried out. Secondly, independent sample *t*-test was used to compare the differences in the dimensions of motivation, attitude and emotional attachment between public and private school students. The independent sample *t*-test was used to compare the average scores of EA dimensions of students in public and private schools, because each dimension represents different aspects of emotional attachment determined by factor analysis, so comparisons were made to test the inter group differences of these dimensions. In order to control the risk of type I errors caused by multiple comparisons, Bonferroni correction was used to adjust the significance level.

Quantitatively, EA was evaluated using a structured scale adapted from established instruments, including Zhang’s Ethnic Identity Scale, Scheve et al.’s multidimensional national identification scale (Zhang, 2011; von Scheve et al., 2014), and thematic insights from the researcher’s preliminary qualitative study. This integrated instrument assessed EA across ten distinct dimensions, such as national identity, cultural identities, attachment to customs, and bloodline connections. The quantitative data allowed for a comparative analysis of EA levels between public and private school groups based on mean scores. As outlined in Appendices A, B, the scale exhibited strong psychometric properties, demonstrating excellent overall reliability (Cronbach’s Alpha = 0.938) and high reliability across all sub-dimensions (ranging from 0.845 to 0.953). The KMO measure confirmed sampling adequacy (KMO = 0.883), affirming the data’s suitability for factor analysis; see section 3.3.2.

### 3.4 Qualitative and quantitative data integration methods

This study uses triangulation to integrate qualitative and quantitative data to enhance the credibility of the research conclusion. Qualitative data include interviews and language portraits. Themes related to emotional attachment and cultural identity are extracted through theme analysis; Quantitative data including questionnaires and exploratory factor analysis (EFA) were used to measure emotional attachment and its dimensions. In the process of integration, qualitative findings are used to explain quantitative trends, and quantitative results in turn verify the universality and structural characteristics of qualitative topics, thus forming a comprehensive analysis framework that verifies and complements each other, and realizing a multidimensional understanding of CHLL language learning experience and identity construction.

## 4 Results

The researcher first transcoded the qualitative data (language portrait narration and interview videos), and then used ATLAS. ti to conduct a typological and thematic analysis to identify, describe, and analyze the language repertoire and EA components. For the quantitative data, the researcher used the SPSS 26.0 program to analyze the participants’ language choices, identities, motivations, and attitudes toward Chinese language learning.

### 4.1 *RQ1*: language input, choices and uses

This sub-section is based on Fishman’s domain and language choice framework. The participants’ language choices are studied in the following domains: (A) family, and (B) the education domain (Fishman, 1972). The results of a multiple-choice survey on individuals’ language choices in various settings and with different forms of communication are analyzed by using the SPSS (26.0) multiple-response analysis. The analysis results are shown in the tables below (Tables 2, 3).

**Table 2:** The statistics of participants’ language choices with grandparents and parents in the family domain.

CO		Father		Mother		Grandparents		Maternal grandparents	
TS		Public	Private	Public	Private	Public	Private	Public	Private
L (%)	1	147 97.35	135 (92.47)	144 (95.36)	137 (93.84)	109 (72.19)	111 (76.03)	103 (68.21)	105 (71.92)
	2	38 (25.17)	22 (15.07)	45 (29.80)	23 (15.75)	8 (5.30)	11 (7.53)	9 (5.96)	6 (4.11)
	3	20 (13.25)	17 (11.64)	24 (15.89)	21 (14.38)	11 (7.28)	7 (4.79)	9 (5.96)	8 (5.48)
	5	23 (15.23)	12 (8.22)	20 (13.25)	18 (12.33)	27 (17.88)	13 (8.90)	12 (7.95)	10 (6.85)
	6	32 (21.19)	16 (10.96)	32 (21.19)	18 (12.33)	39 (25.83)	15 (10.27)	34 (22.52)	13 (8.90)
	7	2 (1.32)	7 (4.79)	6 (3.97)	7 (4.79)	7 (4.64)	5 (3.42)	12 (7.95)	8 (5.48)
	8	17 (11.26)	3 (2.05)	14 (9.27)	2 (1.37)	22 (14.57)	5 (3.42)	21 (13.91)	4 (2.74)
	9	3 (1.99)	/	/	/	2 (1.32)	/	1 (0.66)	/
	10	3 (1.99)	27 (18.49)	2 (1.32)	33 (22.60)	1 (0.66)	31 (21.23)	1 (0.66)	32 (21.92)
	11	/	/	/	1 (0.68)	/	/	/	1 (0.68)
	12	/	/	/	1 (0.68)	/	/	/	/
	13	/	/	/	1 (0.68)	/	/	/	/
	14	/	/	/	/	/	/	/	1 (0.68)
	15	/	/	/	1 (0.68)	/	/	/	/
	16	/	/	/	/	/	/	/	1 (1.59)
	17	/	/	/	/	/	/	/	/
	18	1 (0.66)	3 (2.05)	/	1 (0.68)	14 (9.27)	15 (10.27)	24 (15.89)	22 (15.07)

CO, Communication Objects; TS, Type of Schools; L, Languages.

1 = Mandarin; 2 = English; 3 = Malay; 4 = Tamil; 5 = Cantonese; 6 = Hokkien; 7 = Hakka; 8 = Teochew; 9 = Hainanese; 10 = Foochow; 11 = Hinghua; 12 = Guangxi; 13 = Yunnan; 14 = Puxian; 15 = Iban; 16 = Thai; 17 = Japanese; 18 = Total.

**Table 3:** Percentage of participants’ language choice in the educational domain.

CO		Teacher		Talking about schoolwork with classmates		Gossip with classmates		Talking with the school canteen staff		Talking with school security staff	
TS		Public	Private	Public	Private	Public	Private	Public	Private	Public	Private
L (%)	1	144 (95.36)	144 (98.63)	149 (98.68)	145 (99.32)	149 (98.68)	146 (100.0)	92 (60.93)	143 (97.95)	36 (23.84)	97 (66.44)
	2	124 (82.12)	63 (43.15)	72 (47.68)	21 (14.38)	60 (39.74)	23 (15.75)	55 (36.42)	12 (8.22)	73 (48.34)	43 (29.45)
	3	131 (86.75)	49 (33.56)	75 (49.67)	13 (8.90)	63 (41.72)	15 (10.27)	109 (72.19)	11 (7.53)	138 (91.39)	59 (40.41)
	4	/	/	/	/	/	/	/	/	1 (0.66)	/
	5	1 (0.66)	2 (1.37)	4 (2.65)	5 (3.42)	7 (4.64)	8 (5.48)	2 (1.32)	6 (4.11)	/	/
	6	/	2 (1.37)	3 (1.99)	1 (0.68)	9 (5.96)	3 (2.05)	4 (2.65)	/	/	/
	7	/	/	/	/	1 (0.66)	1 (0.68)	1 (0.66)	1 (0.68)	/	/
	8	/	1 (0.68)	1 (0.66)	1 (0.68)	5 (3.31)	1 (0.68)	1 (0.66)	/	/	/
	9	/	/	1 (0.66)	/	/	/	/	/	/	/
	10	1 (0.66)	3 (2.05)	/	1 (0.68)	/	6 (4.11)	/	4 (2.74)	/	/
	11	/	/	/	/	/	/	/	/	/	2 (1.37)

CO, Communication Objects; TS, Type of Schools; L, Languages.

1 = Mandarin; 2 = English; 3 = Malay; 4 = Tamil; 5 = Cantonese; 6 = Hokkien; 7 = Hakka; 8 = Teochew; 9 = Hainanese; 10 = Foochow; 11 = No communication.

#### 4.1.1 Family domain

Table 1 shows the language choices of the two groups of respondents when interacting with their parents and grandparents in the family domain.

The following are the main language use patterns shared by the two groups of respondents in the family domain: 1. Mandarin is the language of choice for both groups of participants (between 92 and 98%) when speaking to their parents (father and mother) in the family domain; 2. Most participants in both groups demonstrated a substantial decline in the use of Mandarin when communicating with their paternal grandparents (72.19 and 76.03%) and maternal grandparents (68.21 and 71.29%), compared to the language they used when interacting with their parents; 3. The use of English and Malay in conversation with grandparents is also very uncommon in both groups (less than 8% in both groups). This shows that, in both groups, Mandarin has gradually displaced other Chinese varieties in the family domain as generations progressed. This reveals that the frequency of language use when talking to their parents in both school groups is Mandarin > English > other Chinese variant > Malay.

Between the participants in the public and private school groups, there are also several differences in the pattern of language choices used in the family domain: In addition to using Mandarin and other Chinese varieties more frequently while speaking to their parents, participants from the public school group also chose to use English (25.17 and 29.8%) more frequently. On the other hand, the percentage of English used by the participants to talk to their parents in the private school group is 15.70 and 15.75%. When speaking with their grandparents and maternal grandparents, participants in the public school group used other Chinese varieties more frequently than those in the private school group, such as Cantonese, Hokkien, and Teochew. This showed that the students in the private school group had a faster rate of language transfer (from ancestral languages to Mandarin). This scenario might be shaped by the usage of Mandarin as the medium of instruction in their school. Although only one private school in East Malaysia was used for the study’s sample, the results still showed that a significantly greater number of students speak Foochow with their parents or grandparents than Mandarin.

#### 4.1.2 Education domain

In the education domain, the information is focused on language choices and usage when interacting with teachers, classmates during discussions and gossip, cafeteria staff, and school security guards. The statistics for their language choices are shown in Table 2.

Table 3 shows the percentage of language choices performed by the two groups of participants in the school domain. The numerical data demonstrates that: 1. Mandarin is the language of choice for most of the students in the education domain; 2. The usage of other Chinese varieties in educational contexts is typically minimal, especially when interacting with classmates and teachers. Mandarin is used more frequently than English, Malay, and other Chinese varieties. The hierarchical structure of language usage is Mandarin > English > Malay > other Chinese varieties. This hierarchy has validated the influence of Malaysia’s Speak Mandarin Campaign, which has been carried out for the past 40 years. In fact, Mandarin was once less commonly used than regional Chinese varieties among students in Chinese schools. Consequently, non-Mandarin language use in the classroom was strictly forbidden in Chinese schools. Students who use non-Mandarin languages in school are fined and punished by the teacher (Ng, 2017). As a result of this study, Mandarin is now replacing other Chinese varieties as the most common language in Chinese schools.

Upon comprehensive investigation, it becomes apparent that there are divergences in language choices between the public and private school groups. (1) The percentage of students who use Malay in addition to Mandarin when speaking with classmates and teachers is greater in the public-school group. For the private school group, the percentage of students using Mandarin is nearly 100%. This is due to the higher percentage of Malay teachers and students in the public schools, and the higher composition of Chinese teachers and students in private schools. (2) When speaking with school canteen staff, the percentage in Table 4 indicated that the respondents in the public-school group (*N* = 149) used Malay (71.8%) more frequently than Chinese (61.1%), followed by English (36.2%) and other Chinese varieties (5.4%) (i.e., Malay > Chinese > English > other Chinese varieties); however, in the private-school group, Mandarin still dominated (100%), followed by other Chinese varieties (4.8%) and English (3.6%), and then Malay. (3) When speaking with the school security guard, the two groups’ languages of choice are even more different. The public-school group uses Malay the most (91.3%), while Mandarin is the most often used language (88%) in the private-school group. This scenario is determined by the ethnic background of the security guards recruited by the schools. Malay and Indian security guards are commonly found in public schools, whereas Chinese security guards are usually hired in private schools.

**Table 4:** Descriptive statistics and *T*-test of the participants’ attitudes and motivations.

Variables	Group	*N*	*M*	SD	Cohen’s d	95% CI	*t*	df	*p*
Attitudes toward Malaysian Chinese	Public	151	4.22	0.57	0.414	0.107 ~ 0.369	3.568	295.000	< 0.001**
	Private	146	3.98	0.58					
	Total	297	4.10	0.59					
Attitudes toward Mainland Chinese	Public	151	3.57	0.72	0.156	−0.058 ~ 0.304	1.341	295.000	0.181
	Private	146	3.44	0.86					
	Total	297	3.51	0.79					
Attitudes toward the other interracial friends in Malaysia	Public	151	4.06	0.59	0.605	0.245 ~ 0.543	5.199	281.453	< 0.001**
	Private	146	3.66	0.71					
	Total	297	3.86	0.68					
Attitudes toward learning Chinese language (positive)	Public	151	4.28	0.70	0.132	−0.067 ~ 0.250	1.134	295.000	0.258
	Private	146	4.19	0.69					
	Total	297	4.23	0.70					
Attitudes toward learning Chinese language (negative)	Public	151	2.03	0.87	0.060	−0.253 ~ 0.148	−0.516	295.000	0.606
	Private	146	2.09	0.89					
	Total	297	2.06	0.88					
Integrative orientation	Public	151	4.23	0.67	0.232	0.003 ~ 0.310	2.002	295.000	0.046*
	Private	146	4.08	0.67					
	Total	297	4.16	0.68					
Instrumental orientation	Public	151	3.99	0.63	0.256	0.018 ~ 0.313	2.207	295.000	0.028*
	Private	146	3.82	0.66					
	Total	297	3.91	0.65					
Chinese language class anxiety	Public	151	2.29	0.79	0.379	−0.490 ~ −0.121	−3.264	295.000	0.001**
	Private	146	2.60	0.82					
	Total	297	2.44	0.82					
Parental encouragement	Public	151	3.65	0.74	0.367	0.100 ~ 0.431	3.160	295.000	0.002**
	Private	146	3.38	0.71					
	Total	297	3.52	0.73					
Motivational intensity	Public	151	1.94	0.25	0.336	−0.151 ~ −0.029	−2.896	295.000	0.004**
	Private	146	2.03	0.28					
	Total	297	1.98	0.27					
Desire to learn Chinese	Public	151	1.99	0.24	0.037	−0.058 ~ 0.042	−0.315	295.000	0.753
	Private	146	2.00	0.20					
	Total	297	2.00	0.22					

^*^p < 0.05, **p < 0.01.

1 = Strongly disagree; 2 = Disagree; 3 = Neutral; 4 = Agree; 5 = Strongly agree.

### 4.2 *RQ2*: language learning process (attitudes and motivations)

This section discusses and analyzes the motivation and attitudes of the two groups of participants toward learning Mandarin. Eleven areas of attitudes and motivation are examined, including attitudes toward Malaysian Chinese, attitudes toward Chinese from China, attitudes toward other races in Malaysia, attitudes toward learning Mandarin, their motivational tendencies (integrative and instrumental), participants’ classroom anxiety about learning Mandarin, parental encouragement, motivational intensity, and desire to learn Mandarin.

Table 4 presents the overall profile of attitudes and motivations for the two participant groups, including the maximum, minimum, mean, and standard deviation values. A series of independent-samples *t*-tests was conducted, with results reported including M, SD, N, df, *t*, *p*, and Cohen’s *d*. An ANCOVA was also conducted to examine the effect of school type (public vs. private) on attitude and motivation while controlling for grade, gender, and SES proxy (Appendix E). The homogeneity of regression slopes was tested, and no significant School Type × covariate interactions were observed (all *p* > 0.05), permitting the use of ANCOVA without interaction terms. After controlling for covariates, school type had a significant effect on attitude and motivation, *F* (1, 290) = 5.348, *p* = 0.021, *R*^2^ = 0.048. Whereas Grade, gender and SES prox were not significant (*p* > 0.05). Estimated marginal means indicated that public-school students showed higher adjusted motivation scores (*M* = 3.63, SD = 0.35) than private-school students (*M* = 3.47, SD = 0.45).

The first seven dimensions are five-level scale questions, while the latter two are option-based scoring questions. A comparison of the two groups yielded the following results: (1) Both groups have the highest mean values of positive attitudes about learning Mandarin (*M* = 4.28 and *M* = 4.19, respectively) and the lowest mean values of negative attitudes toward learning Mandarin (*M* = 2.03 and *M* = 2.09). (2) Compared to the private school group, the public school group generally has more positive attitudes toward Malaysian Chinese, mainland Chinese, and other racial groups in Malaysia (except the attitudes toward mainland Chinese); the private school group, on the other hand, has moderate attitudes toward these communities (*M* = 3.66–3.98). (3) Both groups’ participants’ motivational tendencies are more oriented toward integration (both scored above 4). When compared to the private school group, the public school group has a higher value of integration and instrumental tendency. (4) The mean score of classroom anxiety about learning Mandarin in the public school group is slightly lower (0.31) than in the private school group, but the level of parental encouragement in the public school group is higher (0.27) than in the private school group. (5) It turns out that the public school group has a higher level of motivation and desire to learn Mandarin than the private school group. The public-school group exhibits a higher level of motivation (*M* = 2.51) than the private-school group. Finally, both groups are found to have a medium level of motivation to learn Mandarin.

### 4.3 *RQ3*: language learning experience through language portraits

To explore the components of participants’ identities and EAs toward Mandarin, 30 subjects from SMK & SMJK and STPC (10 from each type of school) were chosen for a series of in-depth one-to-one online interviews and therefore the language portrait narratives are used as primary data for the thematic analysis, as shown:

#### 4.3.1 My “heart” and “head” are “mandarin”

**Figure 1:**
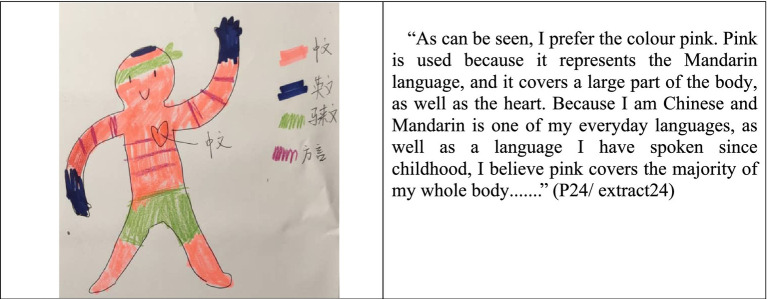
Language portraits and description of P24 (STPC).

Although the layout of the language portraits provided by the 30 participants is varied, almost all of them stated that they did not doubt their identity as Mahua. To identify the symbolic representation of Mandarin using body-related analogies, participants were asked to choose their favorite color on the corresponding part of the given body silhouette. The results revealed that 93% of the participants “draw” Mandarin in the position of the silhouette’s heart, and declared that Mandarin represents their ethnic identity as Mahua, and, thus, the color is compulsory to be filled in the most important part of the body, such as ‘head or heart’. This claim is in accordance with other similar studies, such as those conducted by Busch (2010), which found that important language is mainly located at the head as well as other important parts of the body (such as the heart and chest). Indeed, rather than emphasizing how the represented language is acquired or even the participants’ language proficiency, the language portraits provide an overview of students’ language practices, allowing for the expression of emotions or experiences associated with each language (Busch, 2010). The extracted results are examples from the Language Portraits survey (Figure 1).

#### 4.3.2 Malay: the language for being a true Malaysian

**Figure 2:**
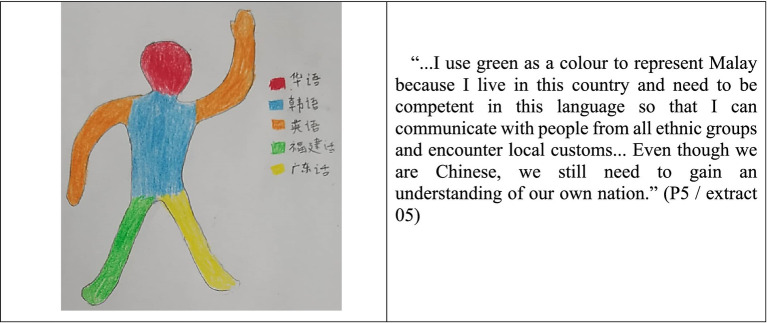
Language portraits and description of P5 (SMJK).

Malay is the second most popular language among the participants studied, with the majority of them (86.7%) colored the legs, body, and other essential parts of the silhouette to represent Malay, while a few people aimed at the upper part of the body, such as ‘heart’ and ‘chest’. They genuinely clarified that Malay is the national language of Malaysia, and being able to communicate in Malay is a symbol of integration and nationalism. Despite Othman et al. (2021) commented that “Chinese students, particularly those with a Mandarin educational background, have a lower sense of national identity” and Baharuddin and Daud (2013) stressed that “the English-educated Chinese students have a lower sense of Chinese identity and are easier to form relationships with other ethnicities than Chinese students educated in Chinese schools”, the results of the language portraits surveys in this study found that the Chinese students with a Mandarin educational background, regardless national SMK/ SMJK or private STPC, are generally having a higher sense of national identity while embracing the national language from young age (Figure 2).

#### 4.3.3 English: an international language that takes me to the world

**Figure 3:**
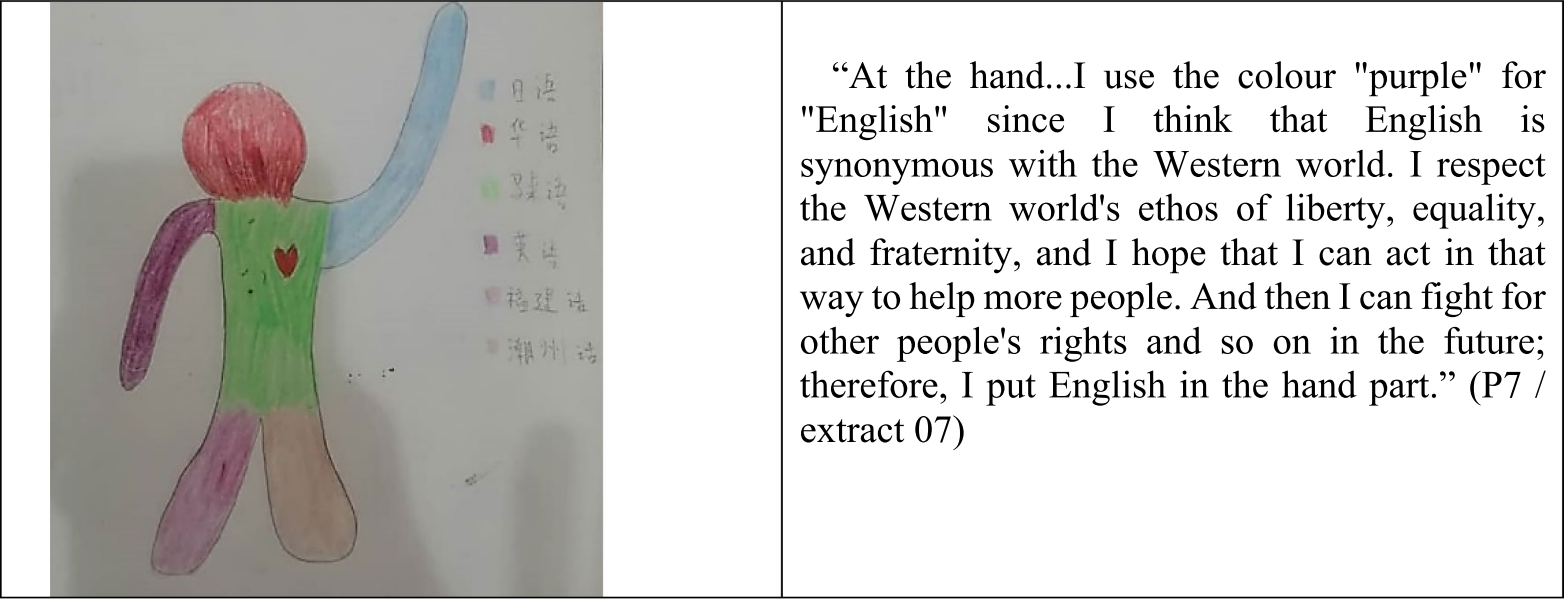
Language portraits and description of P07 (SMJK).

According to the language portraits survey, 50% of respondents preferred English painted on the silhouette’s feet or hands. They claimed that learning English would benefit their future professions by allowing them to “shake hands with more people,” “take them to explore more of the world,” and “develop more opportunities” (Figure 3).

#### 4.3.4 Dialects as a link to the ancestors

**Figure 4:**
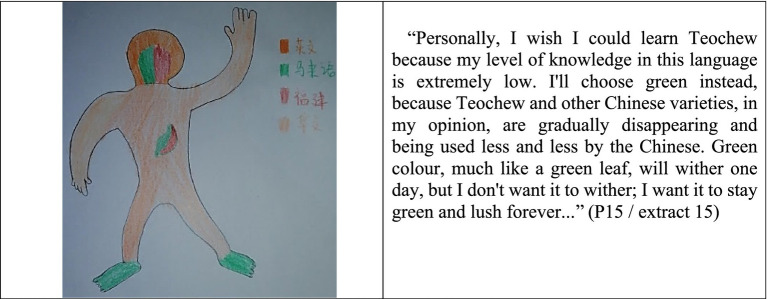
Language portraits and description of P15 (SMK).

About half of the subjects (46.7%) painted their respective Chinese variety on the abdomen of the silhouette, indicating that they considered Chinese varieties such as Teochew, Cantonese, Hakka, and others to be primarily used for family communication, especially when speaking with older generations. They are disappointed if they do not know their ancestral languages well enough to communicate effectively with their grandparents. Some of them regret not having had the opportunity to learn their ancestors’ native tongue. They begin to realize that they are gradually losing their native tongue. Some individuals (P15) have even indicated that they are attempting to learn their ancestral languages and want to do what they can to preserve them (Figure 4).

#### 4.3.5 More languages, more advantages

**Figure 5:**
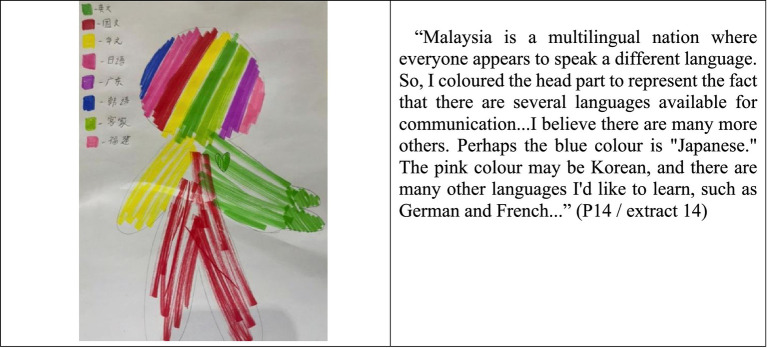
Language portraits and description of P14 (SMK).

In this language portrayal survey, most students (93%) reported their desire to learn specific foreign languages, particularly Japanese and Korean. They all agreed that this would qualify them to engage with global platforms, provide them with a better understanding of other nations’ cultures, and enable them to position themselves effectively in the age of globalization. The following narratives focus on language portraits of other foreign languages (Figure 5).

The results show that the two types of schools are generally consistent in the level of language symbols: the vast majority of students put Mandarin in the “heart” or “head,” as the core of Malaysian Chinese identity; Malay mainly symbolizes national ownership, English is understood as an instrumental language, and dialect symbolizes blood ties. This basic functional stratification of language has no significant difference in different education systems. However, the key differences are reflected in the breadth of emotional attachment and the way of identity integration. Public school students show a more multidimensional emotional structure, which not only emphasizes the connection between Mandarin and Chinese culture, but also expresses strong emotional identity with Malay, showing a more integrated dual identity orientation. In contrast, the emotional focus of participants in private schools is more focused on the internal identity of Chinese culture, and their attitude toward Malay language is more focused on the functional level. In short, public school students tend to adopt the “integrated and extended” identity model, while private school students show a more obvious “cultural cohesion” identity orientation.

### 4.4 *RQ4*: cultural identity construction

This section interprets the findings of the cultural constructs from both qualitative and quantitative perspectives, which are based on thematic and exploratory analyses of aspects related to EA in the two research subject groups.

#### 4.4.1 Qualitative analysis of emotional attachment (EA)

This section focuses on the interpretation of research results based on a thematic analysis of EA-related aspects in the two subject groups. Their EA can be categorized into six groups: daily interaction, literacy/language learning, cultural participation, ethnic identity, “ancestral root” inheritance, and leisure/entertainment.

(1) In daily interaction

Several participants in the interview admitted to having a strong attachment toward Mandarin and other Chinese varieties. They felt more intimate and comfortable while speaking these “HLs” with their family, friends, and community members. The feeling of intimacy enabled them to develop a social sense of belonging. The following remarks from SMJK, SMK, and STPC members indicate this point of view:

*“…because I think Mandarin is very important in my life, I currently use Mandarin to interact with my family; without it, I think many messages would be impossible to convey.” (SMJK P5/extract 05)*

(2) Literacy/Language learning

Among these 30 respondents, 15% of the public school group and 30% of the private school group could speak Mandarin before starting school, and after starting school, they began learning to read and write Chinese characters. The majority of respondents agreed that Chinese should learn Mandarin as it is a significant component of their Chinese identity. They also agreed that the Chinese are responsible for preserving Mandarin, embracing Chinese culture, and engaging in occupations that require proficiency in Mandarin. The following are the quotations:

*“…since I think I am of Chinese descent, so I have to learn Mandarin. I have learned Mandarin since childhood. They all speak Mandarin to me, so I can now speak, listen, read, and write in Mandarin.” (SMJK P3/extract 03)*

(3) Leisure/Entertainment

The EA to Mandarin is demonstrated not only in learners’ daily use of Mandarin but also in their other Mandarin language-related interests and entertainment. Some respondents, for example, stated that, in addition to learning Mandarin in school, they enriched their knowledge of the language by reading Chinese books, searching for online Chinese-related materials in Mandarin, listening to (Mandarin) songs, participating in Mandarin debates, or watching Mandarin TV dramas, etc. They declared:

*“Every month, we have several events at school, and I also take part in a Mandarin essay competition every year. I prepared an essay about Chinese literature during the competition. Yes, the competition requires us to submit our own writing and then publish it in the school’s magazine.” (SMJK P2/extract 02)*

(4) Ethnic identity/Community sustainability

The respondents’ EA Is represented not only by the degree of Mandarin acquisition but also by their identity, ethnicity, background and social context. According to Bond (1993), “Chinese society and culture are expressed in the individual Chinese concepts of food, clothing, housing, language, habits and beliefs (feudal, conservative and traditional cultural content), and the group as the so-called gangs of the geopolitically shared living body” (Ng, 2017). First, in terms of language identity, their views are as follows:

*“I consider Mandarin to be my ‘roots’ since I have studied it for at least ten years, and it represents something like a religious belief.” (STPC P22/extract 22)*

(5) Cultural participation & transmission

Culture (for an ethnic group) is similar to a bloodline that is passed down from one generation to the next. Either “people to culture” or “culture to people” is the fundamental emotion that all humans possess, such a primal emotion is composed of both material and spiritual elements (Busch, 2010; Khairani and Wiradnyana, 2023). As a result, for the Chinese in Malaysia, Malaysian culture contains their most primal emotions and is inextricably linked. The phenomenon of cultural persistence is evident among the new generation of Chinese. Their statements are as follows:

*“Yes, we will go out and buy some decorations for the Chinese New Year. And then we go to see the lion dance on New Year’s Day. I enjoy going shopping for Chinese New Year preparations with my parents. I greatly enjoy this festival because I’m Chinese. For example, I will visit my friends and relatives for the Chinese New Year and attend a Chinese temple to worship deities for blessings. I am drawn to the temple’s carved stone pillars, as well as fortune-telling, to predict my fortune for the upcoming year. This is my annual customary practice.” (SMK P11/extract 11)*

(6) “Ancestral Root” inheritance (Ritual & Folklore purposes)

A desire to discover one’s “ancestral roots” can also be interpreted as an expression of EA. One of the most common ways to transmit EA is through reverence for ancestors. This is how Chinese culture honors their ancestors. The second way is to trace the ancestral descent area. Several respondents reported that they wanted to return to their “homeland” to visit the birthplace of their ancestors and gain a deeper understanding of the reasons for their migration.

*“There are genealogy records in our family. My name is Ting Chiong Yew, and in the genealogy book, ‘Chiong’ is the inherited name, and all of us from the same generation use “Chiong” as our middle name. I believe that Chinese must have Chinese names, and that this is a form of heritage. The genealogical records that I just mentioned are a heritage, as they record our names and can be passed down through generations. As a result, I believe it is very important to have Chinese names. I will name my children using the names from the genealogy book. Thus, it must be passed on to the next generation.” (STPC P26/extract 26).*

#### 4.4.2 Quantitative analysis of EA

This section provides a comprehensive quantitative interpretation and analysis of the level of attachment, as well as the specific elements that constitute it. These attachments are described and analyzed in terms of their internal identities and external manifestations. In terms of internal identities, four dimensions are investigated: national identity, cultural identity (Chinese cultural identity and Malay cultural identity), and traditional Chinese cultural values. For the dimensions of external manifestations, six dimensions have been examined: EA to Malaysian customs, necessities of life (e.g., food, clothing, shelter, and transportation), bloodline and country of origin, the promotion of culture, and customs and traditions.

The main objective of Exploratory Factor Analysis (EFA), a factor analysis approach, is to determine the underlying relationships between measured variables. The purpose of the EFA approach is to enhance the measurement of the factors that were previously generated. Therefore, an EFA was conducted to determine the precise number of components or the relationships between the variables and the relevant dimensions. Additionally, an EFA on the EA dimension was conducted to determine the number of dimensions in EA. The items that are specified in the first stage of the interview are included in the content of the EA questionnaire. These items are extracted using the following criteria, based on the principal component extraction and maximum rotation method, and met the following criteria (Bartlett, 1954; Hair et al., 2011):

(1) The Kaiser-Meyer-Olkin Measure of Sampling Adequacy scale is used to examine the respondents’ values and yielded a value of 0.883, which is greater than the critical value of 0.60 proposed by Kaiser (1974);
(2) Factor eigenvalues greater than 1.0;
(3) Total variance explained equal to or greater than 50% of the common variance. The 10 variables of the variance of the explanation rate after rotation are 10.299, 9.873, 8.974, 8.480, 7.212, 7.079, 5.658, 5.591, 3.550, 2.941%, and the cumulative variance explained after rotation is 69.655%;
(4) All of the research items correspond to a common degree value higher than 0.5, indicating that there is a significant correlation between the research items and the factors, and the factors can effectively extract the information;
(5) The whole Cronbach’s alpha value is 0.938, which for the 10 latitudinal dimensions of EA are 0.948, 0.845, 0.952, 0.928, 0.947, 0.941, 0.936, 0.90, 0.897, and 0.953, which are all greater than the 0.70 cutoff value indicated by Nunnally (Raykov and Marcoulides, 2011).

Finally, Table 5 displays the maximum, minimum, mean, and standard deviation for the remaining 10 dimensions. Overall, the level of EA is significantly greater in the public-school group (*M* = 3.72–4.21) than in the private-school group (*M* = 3.35–3.93). The public-school group has the highest level of EA to Malaysian customs (*M* = 4.21) and the lowest level of EA to bloodline and geography (*M* = 3.72), whereas the private-school group has the highest level of Chinese cultural identification (*M* = 3.93) and the lowest level of EA in terms of cultural promotion (*M* = 3.35).

**Table 5:** Descriptive statistics and *T*-test of participants’ EA.

Variables	Group	*N*	*M*	SD	Cohen’s d	95% CI	*t*	df	*p*
National identity	Public	151	4.03	0.58	0.555	0.197 ~ 0.473	4.778	295.000	< 0.001**
	Private	146	3.69	0.63					
	Total	297	3.86	0.63					
Malaysian cultural identity	Public	151	4.17	0.66	0.624	0.260 ~ 0.561	5.378	295.000	< 0.001**
	Private	146	3.76	0.65					
	Total	297	3.96	0.69					
Chinese cultural identity	Public	151	4.20	0.61	0.412	0.118 ~ 0.413	3.552	295.000	< 0.001**
	Private	146	3.93	0.67					
	Total	297	4.07	0.66					
Tionghua traditional cultural values	Public	151	4.12	0.60	0.490	0.165 ~ 0.452	4.221	295.000	< 0.001**
	Private	146	3.82	0.66					
	Total	297	3.97	0.65					
EA to customs and traditions	Public	151	4.06	0.60	0.596	0.229 ~ 0.513	5.137	295.000	< 0.001**
	Private	146	3.68	0.64					
	Total	297	3.87	0.65					
EA to artistic appreciation	Public	151	3.96	0.67	0.441	0.149 ~ 0.470	3.801	295.000	< 0.001**
	Private	146	3.65	0.74					
	Total	297	3.81	0.72					
EA to basic necessities of life	Public	151	4.04	0.62	0.402	0.115 ~ 0.417	3.465	295.000	< 0.001**
	Private	146	3.78	0.70					
	Total	297	3.91	0.67					
Bloodline and geographical attachment	Public	151	3.68	0.69	0.429	0.137 ~ 0.449	3.693	295.000	< 0.001**
	Private	146	3.38	0.68					
	Total	297	3.53	0.70					
EA embodied in cultural promotion	Public	151	3.87	0.78	0.592	0.314 ~ 0.709	5.104	295.000	< 0.001**
	Private	146	3.35	0.94					
	Total	297	3.61	0.90					
EA to Malaysian customs	Public	151	4.21	0.56	0.755	0.328 ~ 0.614	6.487	280.061	< 0.001**
	Private	146	3.74	0.68					
	Total	297	3.98	0.67					

*p < 0.05; **p < 0.01.

A series of independent-samples *t*-tests was performed, with results reported in terms of M, SD, N, df, t, p, and Cohen’s d. Additionally, an ANCOVA was conducted to examine the effect of school type (public vs. private) on attitudes and motivation while controlling for grade, gender, and SES proxy (see Appendix E). Tests of the homogeneity of regression slopes revealed no significant School Type × covariate interactions (all *p* > 0.10), confirming the suitability of ANCOVA without interaction terms. After adjusting for covariates, school type had a significant effect on attitudes and motivation, *F* (1, 290) = 20.378, *p* < 0.001, R^2^ = 0.011, whereas grade, gender, and SES proxy were nonsignificant (all *p* > 0.05). Estimated marginal means further indicated that public-school students reported higher adjusted motivation scores (*M* = 4.03, SD = 0.52) compared with private-school students (*M* = 3.68, SD = 0.55).

## 5 Discussion

According to the results of RQ1, private school students have a more pronounced language shift, while public school students retain more dialect in their interactions with their grandparents and have a slower rate of language substitution. This phenomenon can be explained in three main ways: first, the institutional influence of the language of instruction. Private schools (especially STPC) tend to use Mandarin as the primary language of instruction, creating an all-Mandarin language environment. This institutional support prompts students to use Mandarin both in and out of school, which further influences home language use habits and accelerates language transfer; second, selective adaptation of home language strategies. Some families from dialectal backgrounds (e.g., Cantonese, Fuzhou) may take the initiative to choose private schools to strengthen their children’s Chinese language proficiency (especially Mandarin) in order to meet the demands of modern society (e.g., college entrance examinations, employment, socializing). To match the school’s language environment, these families may also reduce the use of dialects at home and reinforce Mandarin communication; third, they consider cultural identity and social mobility. Part of the reason why specific communities (e.g., Foochow or Cantonese-speaking families) choose private schools is that Cantonese-speaking families tend to live in the center of West Malaysia, such as Kuala Lumpur and Sibu (in Sarawak, East Malaysia), which, unlike Penang or Johor, are predominantly Chinese and tend to be multilingual and multicultural. Thus, the Chinese in these areas place more emphasis on cultural preservation and investment in linguistic capital. Ironically, however, such choices, although made for cultural transmission purposes, unintentionally accelerate the weakening or marginalization of the dialect due to the school’s strong Mandarin-speaking environment.

These statistics indicate that in the educational domain, the public school group uses Mandarin and Malay on an equal basis, whereas Mandarin remains the most widely used language in the private school group. This is because each of these types of institutions has different educational language policies, resulting in distinct patterns of language choices. The school’s educational language policy has a profound impact on students’ language choices, primarily in terms of the language of instruction, teacher composition, campus communication, and cultural atmosphere. Public schools use Malay as the primary language of instruction, with teachers and administrative staff mainly speaking Malay, and students use Malay more frequently in their daily learning and campus communication. Private Chinese schools, on the other hand, use Mandarin as the primary language of instruction, and teachers and staff are mainly Chinese, making it the preferred language for students in the classroom and daily life. In addition, campus cultural activities reinforce language identity, with public schools placing more emphasis on the national language and fostering a sense of unity, while private schools focus on Chinese culture and the expression of the Chinese language. Parents also make choices based on a school’s language policy, with families who want their children to maintain their mother tongue or improve their Mandarin skills preferring private schools. These institutional differences ultimately led to a clear differentiation in language use, for example, in daily conversations with security guards or cafeteria staff, and language choices were significantly different depending on the type of school. This suggests that educational policies have a direct influence on students’ language behavior and language shift pathways by shaping the language environment.

Regarding the results of RQ2, numerous sociocultural and structural factors interact to produce the phenomena of stronger general language attitudes and motivation, reduced classroom anxiety, and higher parental encouragement among public school pupils. Since students attending public schools are more likely to come from lower-middle-class families with more linguistically diverse backgrounds, they are exposed to a greater variety of Malay and other ethnic groups during their upbringing and depend more on language proficiency for intercultural communication, which makes language acquisition more useful and socially integrative. Furthermore, these families are more likely to emphasize education as a means of achieving upward mobility. They also have more explicit expectations for their children’s Mandarin language proficiency, as well as more active parental supervision and language motivation at home, all of which help to lower kids’ anxiety levels. On the other hand, the bulk of pupils attending private schools came from middle-class and upper-class families, and Mandarin was typically the language of teaching in these institutions. Students’ utilitarian demands and external incentives for Mandarin were reduced due to the more favorable linguistic environment, and instead, their motivation shifted to a more symbolic and identification-based level. Meanwhile, the more competitive environment and higher academic pressure at private schools can make pupils feel more anxious in class. This also explains why some parents choose expensive private education even though public school data is better on several dimensions: they value the purity of the cultural heritage, the safety of the school environment, and the connection of the pathway. Ultimately, the logic of reproducing various socioeconomic classes, educational institutions, and cultural identities in Chinese Malaysian society is reflected more deeply in language choice and learning motivation than simply the rivalry for marks or the language of teaching.

The comparative results of RQ3 show that the differences in education system do not change the basic structure of CHLL at the level of linguistic symbols, but significantly affect the extension of emotional attachment and the way of identity integration. This discovery has important theoretical significance. First, it supports the view that language emotional attachment has “structural stability,” that is, in a multilingual society, the language of the core ethnic group often exists relatively stable as the symbolic center of identity. However, this study further reveals that emotional attachment also has “situational constructiveness”: different educational environments shape the path of students’ identity integration through social interaction patterns and language practice opportunities. Public school students are more likely to develop an integrated dual identity structure in the context of cross ethnic interaction, while private Chinese independent middle school students strengthen the internal identity of ethnic groups in a relatively homogeneous cultural environment. Thus, language is not only a symbolic resource of ethnic identity, but also a socialization result in the context of institutionalized education. Therefore, this study expands the interpretation dimension of language portrait research, from the analysis of a single symbolic space to the dynamic understanding of the relationship between educational context and identity construction, which shows that language emotional attachment has both symbolic stability and social context plasticity.

The results of RQ4 show that in the context of multilingual Malaysia, both public and private schools CHLL build their cultural identity through emotional attachment, and generally strengthen the connection between Mandarin and Chinese identity in daily interaction, language learning and cultural inheritance. However, there are differences between the two types of schools in the path of identity construction. The quantitative results show that public school students have higher emotional attachment in the dimensions of national identity and Malaysian customs, showing an “integrated and extended” mode of integrating national identity on the basis of Chinese culture; Private Chinese independent middle school students are the most dependent on the dimension of Chinese cultural identity, and pay more attention to the internal traditions and cultural continuity of the ethnic group, reflecting the “cultural cohesion” orientation. It can be seen that CHLL’s cultural identity construction not only has the stable core of ethnic culture, but also presents different integration paths under the influence of educational context.

### 5.1 A model of EA for Malaysian CHLLs

Based on the integrated analysis of qualitative and quantitative data, this study constructed the Malaysia CHLLs’ emotional attachment (EA) model to explain how it forms its linguistic and cultural identity in a multilingual society. The comprehensive results show that EA is not a single emotional tendency, but consists of four interrelated dimensions: language input and use, language learning process, language learning experience, and cultural identity construction. These four dimensions interact with each other in structure and jointly shape students’ emotional orientation of Mandarin.

**Figure 6:**
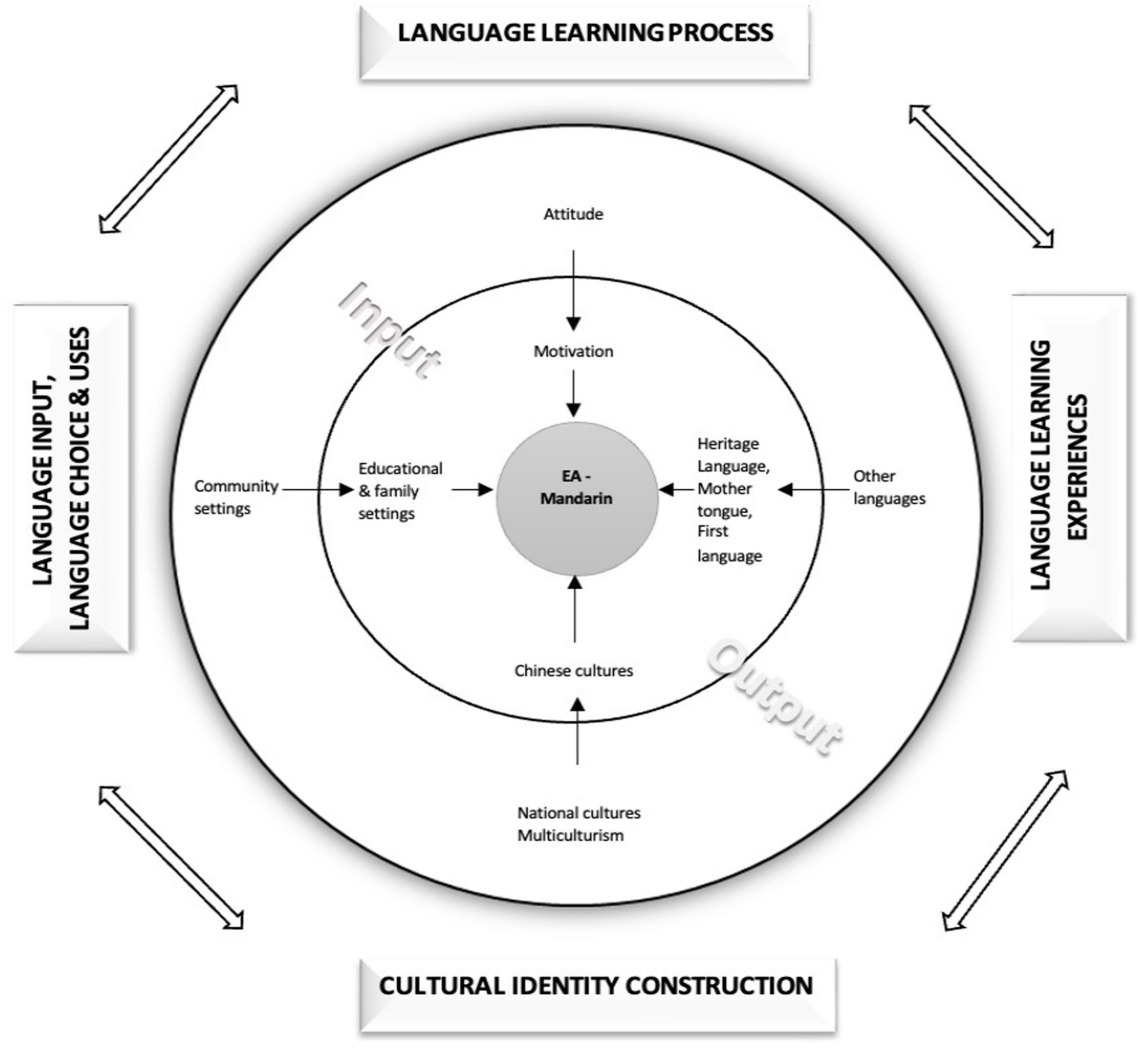
The model of CHLL’ EA development.

The analysis illustrates that EA is composed of four major segments (Figure 6): the language inputs, choices, and uses; the language learning process; the language learning experiences; and the construction of cultural identity. First, language input and use were primarily derived from RQ1 and assessed through the Language Backgrounds and Language Uses questionnaire, which captured students’ exposure to and use of Mandarin in familial, educational, and community contexts. Second, the language learning process was informed by RQ2, which integrated data from the Attitude/Motivation Test Battery (AMTB) questionnaire to reflect students’ motivations, attitudes, and psychological experiences during Mandarin learning. Third, language learning experiences were illustrated through language portraits (RQ3), highlighting students’ perceptions and positioning of Mandarin within a multilingual environment. Finally, the construction of cultural identity was assessed through a combination of interviews and the Identity and EA Questions questionnaire (RQ4), revealing students’ in-depth understanding of the relationship between self-identity and Mandarin in a multicultural context. These four dimensions interact with one another, collectively outlining the structure and content of affective attachment, thereby allowing the construct to be systematically measured and theoretically interpreted.

The model further shows that there is a two-way interaction between these four dimensions. Language input and learning process interact to shape language preference and emotional intensity; The mutual projection of language learning experience and cultural identity construction makes mandarin not only the carrier of emotional expression, but also the symbolic output of identity practice. At the same time, the change of cultural identity will in turn affect the choice and use of language. Thus, EA is a dynamic system, not a static attitude.

In a multilingual environment, Mandarin, Malay and English constitute the core resources of identity negotiation. The results show that Mandarin, as the traditional language of ethnic groups, maintains cultural continuity, while Malay and English provide functional and social integration resources at the national and global levels. The comparison between public and private schools further shows that the educational context affects the extension direction of EA: public school students tend to integrate identity construction, while private school students strengthen ethnic cultural cohesion. However, this difference is reflected in the emotional extension structure, rather than the negation of the core status of Mandarin.

In general, the EA model proposed in this study emphasizes that language emotional attachment has the characteristics of structural stability and situational plasticity. Language, motivation and cultural identity interact continuously in multiple social contexts, which together constitute the dual and even multiple identity framework of CHLL in Malaysia. This model provides a systematic explanation for understanding the maintenance mechanism of heritage language in a multilingual society, and also provides a theoretical basis for language education and cultural policy-making.

### 5.2 Theoretical implications

In terms of its theoretical implications, this study demonstrates that the integrated application of the Psychosocial Model and Overseas Chinese Identity Theory yields a coherent interpretation of its core finding: the variance in students’ attachment to Mandarin across different school types. The Psychosocial Model provides the micro-foundation, emphasizing the determinative role of a learner’s motivation and attitudes in second language acquisition. Conversely, the Theory of Chinese Multiple Identities (Wang, 1988), which anchors Overseas Chinese Identity Theory, supplies the macro-context by conceptualizing overseas Chinese identity as a negotiation between national, cultural, and ethnic dimensions. Consequently, Wang’s (1988) theory illuminates the broader socio-historical circumstances of the Mahua diaspora that shape learner motivations, whereas Gardner’s model offers a framework for interrogating the specific affective drivers (such as integrative and instrumental motivation) involved in learning Mandarin. By using the example from the language portrait findings, which positioned Mandarin in the ‘heart’ and ‘head,’ the interpretation through the lens of Overseas Chinese Identity Theory reveals that this placement serves as a powerful visual metaphor. It signifies that Mandarin is not merely a communication tool but is internalized as the core of their ethnic identity. This finding illustrates what Tan (2004) describes as the ‘symbolic function’ of a heritage language in diaspora communities, where it acts as a boundary marker against assimilation and a vessel for cultural continuity. From a psychosocial perspective, this profound integrative orientation, i.e., the desire to identify with the Chinese-Malaysian in-group is the primary driver of their emotional attachment, overshadowing instrumental concerns.

This study also contributes a critical advancement to the general frameworks prevalent in Anglophone Chinese Heritage Language (CHL) research. That scholarship frequently conceptualizes identity as a negotiation between a monolithic “heritage Chinese identity” and a “national identity” (e.g., American, Canadian), where the primary pressure is assimilation into a dominant monolingual (English) mainstream. Furthermore, CHL is often framed symbolically, as a cultural root to be preserved. Moving beyond this binary and symbolic approach, this study successfully develops a four-dimensional interactive model (language input and choice ➔learning process ➔ learning experience ➔ cultural identity). This model (Figure 6) theorizes EA as a dynamic outcome, continually shaped by the interplay of multiple factors across family, school, societal, and policy levels. Consequently, it reveals that Malaysian CHLLs’ EA and identity are not a static choice between two poles but are negotiated, contextual, and multi-layered, emerging from dynamic interactions across these spheres. This multi-level framework is crucial for understanding the complexity of CHL identity formation and is broadly applicable to other multilingual, multiethnic diaspora contexts where identity is negotiated within complex socio-educational ecosystems.

### 5.3 Significance of the study

This study highlights significant sociocultural and educational implications arising from the comparative results between national and private schools. From a sociocultural perspective, a notable paradox emerges: private Chinese schools (STPCs), established by the community to preserve Chinese culture, are paradoxically accelerating the decline of Chinese ancestral dialects (e.g., Hokkien, Cantonese, Hakka). This occurs through their strict enforcement of standardized Mandarin in education, which systematically facilitates a language shift from regional dialects to Mandarin. In contrast, public schools, the type of schools that often perceived as a threat to Chinese identity because Mandarin is taught only as a subject, inadvertently create an environment where these regional dialects persist longer due to a less immersive Mandarin environment. From an educational standpoint, the differing experiences of students from various school types may deepen social diversification within the Malaysian Chinese community. Public school students typically develop a stronger national identity and daily multilingual skills, leading them to seek higher education at local universities that offer instruction in Malay or English. In contrast, private school students, who possess significant Chinese cultural capital and proficiency in Mandarin, are often drawn to pursue tertiary education in Mandarin-speaking countries such as Taiwan or China. This educational divide ultimately reinforces social diversification, characterized by academic pedigree and the specific type of ‘Chineseness’ that each promotes. The study on EA and identity among Malaysian CHLLs also offers several significant contributions when viewed alongside research on Chinese as a HL in countries like the United States, Canada, and Australia. First, it successfully addresses a critical gap concerning CHL learning and EA within a mainstream educational context (i.e., Malaysia), as opposed to settings outside the formal national education system. Overseas research in contexts such as the US, Canada, and Australia consistently highlights that CHL acquisition primarily occurs in informal or supplementary settings, such as weekend community language schools, family interactions, or university elective courses. The mainstream public education systems in these countries typically do not offer sustained, credit-bearing Mandarin education tailored for heritage learners from elementary through secondary school. In contrast, in Malaysia, Mandarin is taught within the mainstream national education system (e.g., SMJK, STPCs). This study examines how CHLLs develop EA and identity within these institutionalized, state-recognized educational frameworks, a context largely absent in Anglophone CHL research. This provides a crucial comparative perspective on how systemic educational support shapes heritage language attachment, language attitudes, motivation, and identity.

Second, this research underscores the pivotal role of national education policies and school linguistic ecosystems in shaping heritage language attachment. Existing scholarship on CHLL has largely concentrated on micro-level factors such as parental attitudes, home language practices, community school effectiveness, and individual motivation, primarily within overseas contexts. In contrast, this study demonstrates that macro-level educational structures, national policies, and institutional choices within heritage communities themselves can decisively influence linguistic ecologies and, ultimately, personal EA. It specifically reveals the profound impact of educational policy and school environment in mediating EA. For instance, in the context of Chinese language education in Malaysia, the establishment of STPCs aims to safeguard Mandarin language and culture. However, their strict Mandarin-only environments inadvertently accelerate the erosion of other Chinese dialects (e.g., Hokkien, Cantonese) among students, hastening a language shift from ancestral dialects toward standardized Mandarin. Conversely, national-type schools such as SMK and SMJK, which offer limited Mandarin exposure, foster a more linguistically diverse setting. In these environments, Chinese dialects often persist longer among students, co-existing with the strong national identity cultivated through the dominant use of Malay.

Third, by centering the unique Malaysian experience of institutionalized Chinese education, this study provides a critical counterpoint to the well-documented narratives of CHL learning in Western immigrant contexts. It highlights the decisive role of state structures and formal educational pathways in shaping heritage language attachment, arguing that emotional commitment is cultivated at the nexus of family, school, and nation-state. This framework offers significant explanatory power for other multilingual societies and urges global CHL scholars to integrate policy and institutional dimensions more deeply into theories of language maintenance. Furthermore, the study advances a more geographically and institutionally diverse conception of CHLL. It proposes a forward-looking research agenda, suggesting: (i) longitudinal studies to trace the evolution of learner motivation from childhood to adulthood across divergent socio-political contexts; (ii) the use of the Malaysian system as a policy thought experiment for diaspora communities in Anglophone countries; and (iii) the cross-cultural application of the language portrait methodology to investigate where CHLLs in different nations symbolically “place” their heritage and dominant languages.

### 5.4 Limitation of the study

Although this study integrates qualitative and quantitative evidence through mixed methods and compares different education systems, there are still three limitations. First, the sample is mainly from secondary schools in three states of Malaysia. Although it covers public and private schools, the popularization of the results still needs to be cautious. Secondly, the study adopted a cross-sectional design, which failed to capture the long-term changes of emotional attachment and cultural identity. In the future, the stability of the model can be further verified by longitudinal research. Third, qualitative data mainly rely on interviews and language portraits, which may be affected by participants’ self-presentation, but still effectively reveal the core features of language symbols and identity construction. Despite the above limitations, this study still provides a systematic framework for understanding the emotional attachment and cultural identity construction of heritage language learners in a multilingual society, and lays a foundation for subsequent research.

## 6 Conclusion

This study advances existing scholarship in three important ways. First, it integrates the construct of EA with language learning motivation to examine heritage language learning, an angle rarely addressed in studies on Malaysian CHLLs. Second, by comparing students from public (SMK/SMJK) and private Chinese secondary schools (STPC), it highlights the role of schooling context in shaping language attitudes and identity construction, thereby uncovering within-group diversity often overlooked in heritage language research. Third, by applying both sociopsychological models and diaspora identity theory, the study contributes to broader discussions in educational psychology, demonstrating how emotional factors mediate the link between language, culture, and identity in multilingual societies.

To summarize, the results of this study presented in precise detail and are grounded in a structured methodology. The implications of this study have inevitably led to a better understanding of the status of Mandarin among the Chinese community and how they positioned themselves within Malaysia’s multilingual society. Indeed, it has been defined that the Chinese population in Malaysia in the twenty-first century is an ethnic community that is attached to Mandarin for identity manifestation. As Mandarin becomes more important on the global stage, this study contributes to the broadening of Chinese identity theory in Southeast Asia by defining the influence of EA on Mandarin learning. Future studies could broaden the scope of research to include different age groups and compare the EA patterns of grandparents and grandchildren. Additionally, cross-country and cross-regional comparisons can be made, and comparative studies can be conducted with the EA patterns of Chinese from other countries or regions to explore similarities and differences.

## Data Availability

The datasets presented in this article are not readily available because the dataset is restricted to academic use only and may not be used for commercial purposes. Requests to access the datasets should be directed to vanilla131121@163.com.

## Author’s note

Dong Zong refers to the United Chinese School Committees’ Association, established in 1954, which is a private organization dedicated to Chinese education in Malaysia. It comprises the associations of Chinese school boards of directors from various Malaysian states. The Primary School Sixth Form Review (Malay: Ujian Pencapaian Sekolah Rendah; English: Primary School Achievement Test; abbreviated as UPSR) is a nationally standardized examination in Malaysia, jointly conducted annually by the Ministry of Education and the Malaysian Examinations Syndicate (MES). The UPSR is the most important examination for primary school students and is intended to determine the academic qualifications, academic performance and achievement of all Primary 6 students in public and state primary schools and their eligibility for promotion to Form 1 (first year) of secondary school. In data collection, after the questionnaires and interview questions have been approved by JKEUPM (Ethic Committee for Research, UPM), the researcher contacts the coordinators of the selected schools to obtain information, such as the number of classes, students enrolled, class schedules, UPSR scores, etc. The researcher met with the teachers and asked for their engagement to help with this research. They were briefed on this study before assigning a date for on-location research.

## References

[ref1] Abdelhadi M. (2018). Language maintenance factors: reflections on the Arabic language. Asia Pac. J. Adv. Bus. Soc. Stud. 4, 340–351. doi: 10.25275/apjabssv4i1ss9

[ref2] Baharuddin S. A. Daud S. (2013). “Nation, ethnicity, and contending discourse in the Malaysian state,” in State making in Asia, eds. R. Boyd and T.-W. Ngo (Oxfordshire: Taylor and Francis), 134–143.

[ref3] Bartlett M. S. (1954). A note on the multiplying factors for various χ^2^ approximations. J. R. Stat. Soc. Series B Stat. Methodol. 16, 296–298. doi: 10.1111/j.2517-6161.1954.tb00174.x

[ref4] Benrabah M. (2014). “Language and politics in Algeria,” in Language, Ethnic Identity and the state, eds. Safran W. Laponce J. A. (London), 127.

[ref5] Bond M. H. (1993). Emotions and their expression in Chinese culture. J. Nonverbal Behav. 17, 245–262. doi: 10.1007/bf00987240

[ref6] Busch B. (2010). School language profiles: valorizing linguistic resources in heteroglossic situations in South Africa. Lang. Educ. 24, 283–294. doi: 10.1080/09500781003678712

[ref7] Busch B. (2016). “Biographical approaches to research in multilingual settings: exploring linguistic repertoires,” in Researching Multilingualism: Critical and Ethnographic, eds. Martin-Jones M. Martin D. (Oxfordshire, UK: Taylor & Francis), 60–73.

[ref8] Busch B. (2018). The language portrait in multilingualism research: theoretical and methodological considerations. Working Papers Urban Lang. Literacies 236, 2–13.

[ref9] Chen X. Y. (2024). Mandarin Chinese Heritage Language Learning Motivations and Experiences at the post-Secondary level in Canada through Language Ideology (Master’s thesis). Toronto, Canada: University of Toronto.

[ref10] Comanaru R. Noels K. A. (2009). Self-determination, motivation, and the learning of Chinese as a heritage language. Can. Mod. Lang. Rev. 66, 131–158. doi: 10.3138/cmlr.66.1.131

[ref11] Da Wan C. Lee M. N. Sirat M. Heng W. Z. (2020). Identities of Chinese community-based higher education institutions in Malaysia: an exploration study using the concept of ‘roots.’. Int. J. Chin. Educ. 9, 68–88. doi: 10.1163/22125868-12340120

[ref12] Dewaele J.-M. (2010). Emotions in multiple languages. London: Palgrave Macmillan.

[ref9001] Department of Statistics Malaysia. (2026). Demographic Statistic Malaysia, Fourth Quarter, 2025-2026 [Data set]. Department of Statistics Malaysia Official Portal. Available online at: https://www.dosm.gov.my/site/downloadrelease?id=demographic-statistic-malaysia-fourth-quarter-2025&lang=English&admin_view=

[ref13] Fishman J. A. (1972). “Domains and the relationship between micro and macro sociolinguistics,” in Directions in Sociolinguistics: The Ethnography of Communication, eds. Gumperz J. Hymes D. (New York: Holt, Rinehart and Winston), 435–453.

[ref9002] Gardner R. C. Lambert W. E. (1972). Attitudes and motivation in second language learning. Rowley, MA: Newbury House.Available online at: https://eric.ed.gov/?id=ED081270

[ref14] Gardner R. Lalonde R. Moorcroft R. (1985). The role of attitudes and motivation in second language learning: correlational and experimental considerations. Lang. Learn. 35, 207–227. doi: 10.1111/j.1467-1770.1985.tb01025.x

[ref15] Gomaa Y. A. (2011). Language maintenance and transmission: the case of Egyptian Arabic in Durham, UK. Int. J. Engl. Linguist. 1, 26–53. doi: 10.5539/ijel.v1n1p46

[ref16] Hair J. F. Ringle C. M. Sarstedt M. (2011). PLS-SEM: indeed a silver bullet. J. Mark. Theory Pract. 19, 139–152. doi: 10.2753/mtp1069-6679190202

[ref17] Hatoss A. Sheely T. (2009). Language maintenance and identity among Sudanese-Australian refugee-background youth. J. Multiling. Multicult. Dev. 30, 127–144. doi: 10.1080/01434630802510113

[ref18] Ho P. Y. Chew F. P. Thock K. P. (2015). Mother tongue education and ethnic identity of Malaysian Chinese secondary school students. In Proceedings of the Annual International Conference on Management and Technology in Knowledge, Service, Tourism & Hospitality 2015 (SERVE 2015) (pp. 1–2). Bandung, Indonesia.

[ref7001] How S. Y. Chan S. H. Abdullah A. N. (2015). Language vitality of Malaysian languages and its relation to identity. GEMA Online Journal of Language Studies, 15, 119–139. doi: 10.17576/gema-2015-1502-08

[ref19] Huang H. Liao W. Y. (2023). Maintaining a minor language or a heritage language? A case study of maintaining Chinese with preteenagers in Australian interlingual families. Int. J. Bilingual Educ. Bilingual. 27, 360–373. doi: 10.1080/13670050.2023.2173519

[ref20] Kaiser H. F. (1974). An index of factorial simplicity. Psychometrika 39, 31–36. doi: 10.1007/bf02291575

[ref21] Kelleher A. M. (2010). Policies and Identities in Mandarin Education: The Situated Multilingualism of University-level Heritage Language Learners. ProQuest LLC eBooks (pp. 110–120). Available online at: https://eric.ed.gov/?id=ED523326 (Accessed July 12, 2025).

[ref22] Khairani L. Wiradnyana K. (2023). From ethnic genealogical folklore to the power and legitimacy of traditional society. Eduvest J. Universal Stud. 3, 1098–1115. doi: 10.59188/eduvest.v3i6.837

[ref23] Khoo J. (1999). The straits Chinese: a cultural history. Available online at: http://ci.nii.ac.jp/ncid/BA45771324 (Accessed July 9, 2025).

[ref24] Kondo-Brown K. (2003). Heritage language instruction for post-secondary students from immigrant backgrounds. Herit. Lang. J. 1, 1–25. doi: 10.46538/hlj.1.1.1

[ref25] Liao C. (2020). Family language policy in a Hakka community in Sabah, Malaysia. J. Mod. Lang. 30, 122–141. doi: 10.22452/jml.vol30no1.4

[ref26] Liu H. (2025). Emotion and identity in an interconnected world: a case study of two Chinese heritage language learners in the United States. Herit. Lang. J. 22, 1–33. doi: 10.1163/15507076-bja10036

[ref27] Masgoret A. Gardner R. C. (2003). Attitudes, motivation, and second language learning: a meta-analysis of studies conducted by Gardner and associates. Lang. Learn. 53, 123–163. doi: 10.1111/1467-9922.00212

[ref28] Montrul S. (2016). The Acquisition of Heritage Languages. Cambridge: Cambridge University Press.

[ref29] Ng P. C. L. (2017). A study of attitudes of dialect speakers towards the speak mandarin campaign in Singapore. SpringerBriefs in Linguistics, Singapore, 25–30.

[ref30] Noels K. A. (2005). Orientations to learning German: heritage language learning and motivational substrates. Can. Mod. Lang. Rev. 62, 285–312. doi: 10.3138/cmlr.62.2.285

[ref31] Othman I. W. Esa M. S. Bakar A. L. A. Mokhtar S. (2021). The relevance of nationhood knowledge in Malaysian studies courses: a conveyance for national unity and an integration of university students’ identity. J. Inf. Syst. Technol. Manag. 6, 01–20. doi: 10.35631/jistm.623001

[ref32] Panicacci A. (2019). Do the languages migrants use in private and emotional domains define their cultural belonging more than the passport they have? Int. J. Intercult. Relat. 69, 87–101. doi: 10.1016/j.ijintrel.2019.01.003

[ref33] Park H. Tsai K. M. Liu L. L. Lau A. S. (2012). Transactional associations between supportive family climate and young children’s heritage language proficiency in immigrant families. Int. J. Behav. Dev. 36, 226–236. doi: 10.1177/0165025412439842

[ref34] Pavlenko A. (2005). Emotions and Multilingualism. Cambridge: Cambridge University Press.

[ref35] Prasad G. L. (2014). Portraits of plurilingualism in a French international school in Toronto: exploring the role of visual methods to access students’ representations of their linguistically diverse identities. Can. J. Appl. Linguist. 17, 51–77. Available online at: https://journals.lib.unb.ca/index.php/CJAL/article/view/22126 (Accessed March 19, 2026).

[ref36] Rasdi M. T. M. (2020). Rethinking Malaysia: Politics, Extremism and Education. Kuala Lumpur: Strategic Information and Research Development Centre.

[ref37] Raykov T. Marcoulides G. A. (2011). Introduction to psychometric theory (pp. 37–58). New York: Routledge.

[ref38] Rosiak K. (2023). The role of language attitudes and ideologies in minority language learning motivation: a case study of polish migrants’ (de)motivation to learn welsh. Eur. J. Appl. Linguist. 11, 26–52. doi: 10.1515/eujal-2021-0018

[ref39] Samuel M. Tee M. Y. (2013). “Malaysia: Ethnocracy and education,” in Education in South-East Asia, ed. Lorraine P. S.. 1st ed (London: Bloomsbury Academic), 137–155.

[ref40] Segawa N. (2019). National Identity, Language and Education in Malaysia: Search for a middle ground between Malay Hegemony and Equality. Oxfordshire: Routledge.

[ref41] Soares C. T. Duarte J. De Meij M. G. (2020). ‘Red is the colour of the heart’: making young children’s multilingualism visible through language portraits. Lang. Educ. 35, 22–41. doi: 10.1080/09500782.2020.1833911

[ref42] Stoessel S. (2002). Investigating the role of social networks in language maintenance and shift. Int. J. Sociol. Lang. 2002, 93–131. doi: 10.1515/ijsl.2002.006

[ref43] Sua T. Y. Santhiram R. (2017). “Race-based policies and practices in Malaysia’s education system,” in Education in Malaysia: Education in the Asia-Pacific region: Issues, Concerns and Prospects, eds. Samuel M. Tee M. Symaco L., vol. 39 (Singapore: Springer).

[ref44] Süleyman K. (2024). Sociopsycholinguistic perspectives: language use and social dynamics among Kurdish speakers in Turkey. Psycholinguistics 36, 147–170. doi: 10.31470/2309-1797-2024-36-2-147-170

[ref45] Tan C. B. (2004). Chinese Overseas: Comparative Cultural Issues. Hong Kong: Hong Kong University Press.

[ref46] Tan Y. S. (2011). Democratization of secondary education in Malaysia: attitudes towards schooling and educational aspirations. Asia Pac. J. Educ. 31, 1–18. doi: 10.1080/02188791.2011.544055

[ref47] Tan Y. S. Teoh H. S. (2014). The development of Chinese education in Malaysia, 1952–1975: political collaboration between the Malaysian Chinese association and the Chinese educationists. Hist. Educ. 44, 83–100. doi: 10.1080/0046760x.2014.959073

[ref48] von Scheve C. Kozłowska M. Ismer S. Beyer M. (2014). “National identification: a multidimensional scale based on a three-country study,” in Berlin Studies on the Sociology of Europe (BSSE, 29), (Berlin: Freie Universität Berlin, FB Politik- undSozialwissenschaften, Institut für Soziologie Arbeitsbereich Makrosoziologie), 3–32.

[ref49] Wang G. W. (1988). “The study of Chinese identities in Southeast Asia,” in Changing Identities of the Southeast Asian Chinese, eds. Wang G. W. Cushman (Hong Kong: Hong Kong University Press), 1–22.

[ref50] Wang X. M. (2018). Huayu, Chinese education and identity: the Malaysian experience. Chin. J. Lang. Policy Plan. 3, 49–57.

[ref51] Wen X. H. (2024). Chinese heritage language motivation: a study of motivation development in a multicultural context. Front. Psychol. 15:1452547. doi: 10.3389/fpsyg.2024.1452547, 39534475 PMC11554449

[ref52] Williams M. Burden R. Lanvers U. (2002). ‘French is the language of love and stuff’: student perceptions of issues related to motivation in learning a foreign language. Br. Educ. Res. J. 28, 503–528. doi: 10.1080/0141192022000005805

[ref53] Wong S. L. Lee H. P. (2003). “The Chinese language movement and national identity in Malaysia,” in Nationalism and Education in Asia, eds. Kang S. M. Smith T. C. (New York, NY: Routledge Falmer), 209–223.

[ref54] Yao M. (2021). Chinese diaspora, Huayu community and Huayu inheritance in Malaysia. Chin. J. Lang. Policy Plan. 6, 11–18.

[ref55] Zhang S. F. (2011). The Development of the L1 Education of the Chinese Immigrants in the Philippine Chinese Immigration in the vision of the Ethnic Cultural Identity (Doctoral Dissertation, Fujian Normal University, China). Available online at: https://kns.cnki.net/kcms2/article/abstract?v=gpYlZH45TFlhWhfYvcmJR6GzkXSHMz9hSkU80U-T7kl-deEC-LKwCIFfZRel0HadMmPjj6u84aA76DFHV2LnEOJIyEhLfs5aHX-VY7f4gSopvUxIrQt1aORgRj8rnlvIJKzV5G3DDtwEzABIoe0BOqK_ZtwyahyeWo8qzcT4ujgrRwrkwD76zTPacHKSi3WO&uniplatform=NZKPT&language=CHS (Accessed June 22, 2025).

[ref56] Zhang J. (2015). Language attitudes and identities in multilingual China: a linguistic ethnography. Int. J. Bil. Educ. Bil. 19, 471–474. doi: 10.1080/13670050.2015.1055934

[ref57] Zhang H. C. Gong Y. Zhang L. Y. Gao X. S. (2024). Teaching and learning Chinese as a heritage language (CHL). Circ. Lingüíst. Apl. Comun. 101, 95–109. doi: 10.5209/clac.100073

[ref58] Zheng L. X. (2023). An analysis of the impact of Chinese heritage language on the identity of the Chinese community in the United States. Proceedings of the 2022 International Conference on Science Education and Art Appreciation (SEAA 2022). Available online at: https://www.atlantis-press.com/proceedings/seaa-22/125976982 (Accessed September 16, 2025).

[ref59] Zhou Q. S. (2016). A review of research on language and identity in China. Chin. J. Lang. Policy Plan. 1, 72–79.

[ref60] Zou C. Y. (2018). Maintenance of Chinese language in Malaysia, from language ideology to practical efforts: a literature review study. Lang. Policy Lang. Educ. 21-32+116–117.

