# Supplementary material for: Portraying identity Mahua through emotional attachment toward Chinese Mandarin among Malaysian Chinese heritage language learners
Source: Front Psychol. 2026 Apr 1;17:1681427. doi: 10.3389/fpsyg.2026.1681427 (PMC13079624; doi:10.3389/fpsyg.2026.1681427)
Supplement: Supplementary file 1 [file Data_sheet_1.pdf]

Appendix

A. Reliability Statistics of Identity & Emotional Attachment Scale

Variables	Items	Corrected Item-Total Correlation	Cronbach's Alpha if Item Deleted	Cronbach's Alpha	Total Cronbach's Alpha
National identity	J1	0.577	0.949	0.948	0.938
	J2	0.778	0.942		
	J3	0.797	0.942		
	J4	0.818	0.941		
	J5	0.740	0.944		
	J6	0.707	0.945		
	J7	0.861	0.940		
	J8	0.819	0.941		
	J9	0.760	0.943		
	J10	0.748	0.943		
	J11	0.744	0.944		
	J12	0.713	0.945		
Malaysian Cultural identity	K1	0.731	0.783	0.845	
	K2	0.570	0.856		
	K3	0.695	0.797		
	K4	0.745	0.776		
Chinese Cultural identity	L1	0.814	0.947	0.952	
	L2	0.865	0.945		
	L3	0.753	0.949		
	L4	0.824	0.946		
	L5	0.715	0.951		
	L6	0.724	0.951		
	L7	0.859	0.945		
	L8	0.863	0.944		
	L9	0.775	0.948		
	L10	0.786	0.948		
Tionghua traditional cultural values	M1	0.832	0.910	0.928	
	M2	0.864	0.905		
	M3	0.855	0.907		
	M4	0.689	0.928		
	M5	0.681	0.930		
	M6	0.832	0.910		
EA to Customs and Traditions	N1	0.797	0.941	0.947	
	N2	0.808	0.941		
	N3	0.830	0.940		
	N4	0.814	0.940		
	N5	0.607	0.947		
	N6	0.776	0.942		
	N7	0.723	0.943		
	N8	0.731	0.943		
	N9	0.697	0.944		
	N10	0.725	0.943		
	N11	0.637	0.946		
	N12	0.696	0.944		
	N13	0.807	0.941		
EA to Artistic Appreciation	O1	0.711	0.938	0.941	
	O2	0.786	0.933		
	O3	0.751	0.935		
	O4	0.776	0.934		
	O5	0.840	0.929		
	O6	0.834	0.930		
	O7	0.758	0.935		

	O8	0.850	0.928	
EA to Clothing, Food, Housing and Transport	P1	0.815	0.924	0.936
	P2	0.759	0.928	
	P3	0.692	0.933	
	P4	0.780	0.927	
	P5	0.834	0.923	
	P6	0.872	0.921	
	P7	0.868	0.920	
	P8	0.601	0.940	
Bloodline and geographical attachment	Q1	0.824	0.873	0.900
	Q2	0.806	0.874	
	Q3	0.684	0.888	
	Q4	0.669	0.890	
	Q5	0.716	0.885	
	Q6	0.662	0.892	
	Q7	0.606	0.897	
EA embodied in Cultural Promotion	R1	0.771	0.875	0.897
	R2	0.805	0.846	
	R3	0.814	0.838	
EA to Malaysian customs	S1	0.829	0.947	0.953
	S2	0.828	0.947	
	S3	0.856	0.945	
	S4	0.876	0.944	
	S5	0.872	0.945	
	S6	0.799	0.948	
	S7	0.801	0.948	
	S8	0.734	0.952	

B. KMO and Bartlett's Test of Identity & Emotional Attachment Scale

Kaiser-Meyer-Olkin Measure of Sampling Adequacy.		0.883
Bartlett's Test of Sphericity	Approx. Chi-Square	20320.180
	df	3160
	Sig.	0.000

C. Total Variance Explained of Emotional Attachments

Component	Initial Eigenvalues			Extraction Sums of Squared Loadings			Rotation Sums of Squared Loadings		
	Total	% of Variance	Cumulative %	Total	% of Variance	Cumulative %	Total	% of Variance	Cumulative %
1	14.289	17.861	17.861	14.289	17.861	17.861	8.239	10.299	10.299
2	7.503	9.379	27.240	7.503	9.379	27.240	7.898	9.873	20.172
3	6.855	8.568	35.809	6.855	8.568	35.809	7.179	8.974	29.145
4	5.679	7.099	42.907	5.679	7.099	42.907	6.784	8.480	37.625
5	5.543	6.928	49.836	5.543	6.928	49.836	5.770	7.212	44.837
6	4.090	5.112	54.948	4.090	5.112	54.948	5.663	7.079	51.916
7	3.916	4.895	59.843	3.916	4.895	59.843	4.526	5.658	57.574
8	3.353	4.191	64.035	3.353	4.191	64.035	4.472	5.591	63.164
9	2.546	3.183	67.217	2.546	3.183	67.217	2.840	3.550	66.714
10	1.950	2.437	69.655	1.950	2.437	69.655	2.352	2.941	69.655

11	1.000	1.250	70.905
12	0.902	1.128	72.033
13	0.879	1.099	73.131
14	0.854	1.067	74.198
15	0.809	1.012	75.210
16	0.781	0.977	76.187
17	0.739	0.923	77.110
18	0.701	0.876	77.986
19	0.682	0.852	78.838
20	0.660	0.825	79.663
21	0.622	0.778	80.441
22	0.594	0.743	81.183
23	0.581	0.726	81.909
24	0.565	0.707	82.616
25	0.536	0.670	83.286
26	0.527	0.658	83.944
27	0.509	0.637	84.581
28	0.478	0.598	85.179
29	0.466	0.582	85.762
30	0.449	0.561	86.323
31	0.425	0.532	86.855
32	0.418	0.523	87.377
33	0.402	0.503	87.880
34	0.394	0.492	88.372
35	0.374	0.467	88.839
36	0.366	0.457	89.296
37	0.356	0.445	89.742
38	0.347	0.434	90.175
39	0.338	0.422	90.598
40	0.329	0.412	91.009
41	0.325	0.406	91.415
42	0.318	0.398	91.814
43	0.309	0.386	92.200
44	0.304	0.380	92.580
45	0.291	0.363	92.943
46	0.284	0.355	93.298
47	0.272	0.340	93.638
48	0.257	0.321	93.959
49	0.254	0.318	94.276
50	0.241	0.302	94.578
51	0.238	0.298	94.876
52	0.234	0.292	95.168
53	0.229	0.286	95.454
54	0.213	0.267	95.721

55	0.209	0.262	95.982
56	0.204	0.255	96.238
57	0.195	0.244	96.482
58	0.186	0.232	96.714
59	0.179	0.223	96.937
60	0.177	0.221	97.158
61	0.167	0.209	97.367
62	0.161	0.202	97.569
63	0.152	0.190	97.759
64	0.150	0.187	97.946
65	0.144	0.179	98.126
66	0.135	0.169	98.295
67	0.135	0.168	98.463
68	0.127	0.159	98.622
69	0.119	0.149	98.771
70	0.115	0.144	98.915
71	0.113	0.141	99.056
72	0.108	0.136	99.191
73	0.106	0.132	99.323
74	0.100	0.125	99.448
75	0.091	0.113	99.562
76	0.085	0.106	99.668
77	0.079	0.098	99.766
78	0.070	0.087	99.853
79	0.064	0.080	99.933
80	0.053	0.067	100.000

D. Rotated Component Matrix of Emotional Attachments

Items	Component									
	1	2	3	4	5	6	7	8	9	10
J1		0.634								
J2		0.798								
J3		0.816								
J4		0.833								
J5		0.786								
J6		0.743								
J7		0.880								
J8		0.844								
J9		0.780								
J10		0.761								
J11		0.753								
J12		0.744								
K1									0.831	

K2									0.730
K3									0.813
K4									0.855
L1			0.855						
L2			0.893						
L3			0.783						
L4			0.854						
L5			0.753						
L6			0.741						
L7			0.869						
L8			0.872						
L9			0.826						
L10			0.829						
M1								0.873	
M2								0.894	
M3								0.878	
M4								0.750	
M5								0.747	
M6								0.868	
N1	0.828								
N2	0.859								
N3	0.862								
N4	0.843								
N5	0.632								
N6	0.825								
N7	0.745								
N8	0.771								
N9	0.710								
N10	0.763								
N11	0.672								
N12	0.711								
N13	0.843								
O1					0.760				
O2					0.840				
O3					0.790				
O4					0.821				
O5					0.848				
O6					0.847				
O7					0.805				
O8					0.862				
P1						0.838			
P2						0.775			
P3						0.700			
P4						0.812			

P5						0.845				
P6						0.865				
P7						0.881				
P8						0.660				
Q1							0.878			
Q2							0.862			
Q3							0.762			
Q4							0.746			
Q5							0.787			
Q6							0.732			
Q7							0.683			
R1										0.816
R2										0.834
R3										0.868
S1				0.850						
S2				0.852						
S3				0.880						
S4				0.886						
S5				0.881						
S6				0.828						
S7				0.815						
S8				0.768						
S9				0.743						

*J1-J12=National identity, K1-K4=Malaysian cultural identity, L1-L10=Chinese cultural identity, M1-M6=Tionghua traditional values, N1-N13= Customs and traditions, O1-O8=Artistic appreciation, P1-P8=Traditional attire, food, sites in China, Q1-Q7= Bloodline and regional affinities attachment, R1-R3=Cultural promotion, S1-S9=Malaysian customs

E. Results of the attitude and motivation's covariance analysis

Source of variance	Sum of squares	df	Mean square	F	p
Intercept	90.524	1	90.524	555.510	< .001**
Type of Secondary school	0.871	1	0.871	5.348	0.021*
Age	0.102	1	0.102	0.626	0.429
Sex	0.020	1	0.020	0.126	0.723
Your Current Year of study/ Grade	0.352	1	0.352	2.159	0.143
Your father's level of education	0.052	1	0.052	0.317	0.574
Your mother's level of education	0.011	1	0.011	0.070	0.791
Residual	47.258	290	0.163		

Note: R^2= 0.048

* p<0.05 ** p<0.01

Type of Secondary school

Group	M	SD	n
Public school	3.63	0.35	151
Private school	3.47	0.45	146

F. Results of the EA's covariance analysis

Source of variance	Sum of squares	*df*	Mean square	*F*	*p*
Intercept	105.289	1	105.289	365.646	< .001**
Type of Secondary school	5.868	1	5.868	20.378	0.000**
Age	0.098	1	0.098	0.340	0.560
Sex	0.302	1	0.302	1.049	0.307
Your Current Year of study/ Grade	0.302	1	0.302	1.049	0.307
Your father's level of education	0.327	1	0.327	1.136	0.287
Your mother's level of education	0.078	1	0.078	0.271	0.603
Residual	83.506	290	0.288		

Note: R^2 = 0.110

* p<0.05 ** p<0.01

Type of Secondary school

Group	M	SD	n
Public school	4.03	0.52	151
Private school	3.68	0.55	146
